# Interventions with Digital Tools for Mental Health Promotion among 11–18 Year Olds: A Systematic Review and Meta-Analysis

**DOI:** 10.1007/s10964-023-01735-4

**Published:** 2023-02-08

**Authors:** Michaela Wright, Franziska Reitegger, Herald Cela, Andrea Papst, Barbara Gasteiger-Klicpera

**Affiliations:** 1 Research Center for Inclusive Education (RCIE), University of Graz, Graz, Austria; 2 Institute of Education Research and Teacher Education, University of Graz, Graz, Austria; 3 Institute of Psychology, University of Graz, Graz, Austria

**Keywords:** Prevention, Well-being, Protective factors, Anxiety, Meta-analysis, Digital programs

## Abstract

The availability of digital tools aiming to promote adolescent mental health is rapidly increasing. However, the field lacks an up-to-date and focused review of current evidence. This study thus looked into the characteristics and efficacy of digital, evidence-based mental health programs for youth (11–18 years). The selection procedure followed the Preferred Reporting Items for Systematic Review and Meta-Analyses (PRISMA) guidelines and resulted in 27 eligible studies. The high heterogeneity of the results calls for careful interpretation. Nevertheless, small, but promising, effects of digital tools were found with respect to promoting well-being, relieving anxiety, and enhancing protective factors. Some important factors influencing overall efficacy include the given setting, the level of guidance and support, and the adherence to the intervention.

## Introduction

Between 10 and 20% of children and adolescents are affected by mental health issues worldwide (Kieling et al., 2011). Increasingly, digital technologies, such as mobile apps or web-delivered programs, are being used to meet the needs of mental health promotion within this age group (Bergin et al., 2020). Thus, the range of digital tools aiming to promote mental health is growing rapidly, and an increasing number of researchers are reporting on their potential and value (Lucas-Thompson et al., 2019; Sommers-Spijkerman et al., 2021). However, an up-to-date and focused review of current evidence is still lacking in the field. The present study thus aims to systematically review and analyze the availability and effectiveness of digital tools for mental health promotion among 11–18 year olds.

Although prevention and intervention programs can be implemented at any point across an individual’s lifespan, they are most effective when provided early, or at the time of disorder emergence (Polanczyk et al., 2015; Solmi et al., 2022). For approximately half of the individuals suffering from a mental disorder, the onset of illness occurs during adolescence, and in more than a third, the disorder emerges by the age of 14 (Solmi et al., 2022). This is especially relevant for neurodevelopmental disorders as well as for anxiety and fear-related disorders. For example, 51.8% of those affected developed anxiety/fear-related disorders before the age of 18 (Solmi et al., 2022). During this transition period from childhood to adulthood, young people face a number of social, physical and emotional challenges (e.g., relating to academic expectations, physical changes, identity and role development) which make them highly vulnerable (Byrne et al., 2007).

The estimated worldwide prevalence of mental disorders was reported to be 13.4% (CI 95% 11.3–15.9) for this population (Polanczyk et al., 2015). Disorder prevalence was highest for anxiety at 6.5% of the population, 2.6% for depressive disorder, 3.4% for attention-deficit hyperactivity, and 5.7% for disruptive disorders (Polanczyk et al., 2015). In addition, mental disorders among adolescents have increased in recent years (Atladottir et al., 2015; Steffen et al., 2018), with the increase being most notable with respect to developmental and mood disorders (Steffen et al., 2018). Suffering from mental illness not only affects the quality of daily life. It has also been found to decrease life expectancy by up to 10–15 years (Walker et al., 2015). This is true not only for those severe mental health problems exhibiting low rates of prevalence, such as psychosis, but also for milder mental disorders exhibiting higher prevalence rates, such as anxiety and depression (Walker et al., 2015).

Mental health encompasses not only one’s internal experience, but also shapes the way one connects and interacts with the external world. Therefore, an understanding of mental health needs to reflect the broad diversity of human experience (Galderisi et al., 2015). Mental health may be described as a “dynamic state of internal equilibrium” (Galderisi et al., 2015, pp. 231–232), i.e., as a malleable state that affects how we relate to ourselves and others. Factors such as cognitive and social skills, the ability to empathize, resilience, self-awareness, self-expression and regulation of emotions, all contribute to mental health in varying degrees and interact dynamically (Galderisi et al., 2015). The complexity and multifaceted nature of the phenomenon is also mirrored in the wide range of methods and instruments used to measure and promote mental health. Though the concept remains difficult to circumscribe, especially the difficulty of distinguishing conceptually between well-being and mental health (Galderisi et al., 2015), in a preliminary literature search, several domains closely connected to mental health and to efforts to enhance it were found: mental health literacy, well-being, resilience, mindfulness, stress management, relaxation, help-seeking behavior and positive psychology.

Since 2020, governmental policies within the context of the COVID-19 pandemic, such as enforced isolation or school closures, have most likely increased the strain on young people’s well-being and raised the risk of developing mental health problems. The frequency of lower health-related quality of life, and higher anxiety levels is now higher than that reported before COVID-19, especially among those with low socio-economic status, a migration background, or limited living space (Ravens-Sieberer et al., 2021). Looking at the alarming number of young people suffering from mental health issues (Atladottir et al., 2015) and the added stressors caused by the pandemic (Ravens-Sieberer et al., 2021), the urgency needed in providing support for this group is clearly evident. Hence, preventing mental disorders and promoting mental health in youth continues to be a main concern in health policies and strategy reports, both on the European (WHO, 2013), and global level (WHO, 2004, 2017).

The ongoing advances in technology mean that more and more mental health prevention programs may be provided successfully, either partly or fully, through digital media (Kaess et al., 2021, 2021; Mrazek et al., 2019). As barriers to mental health services increased during the pandemic (due to lockdowns and restrictions), the advantages of choosing a digital mode of delivery have become manifold, e.g., cost-effectiveness, anonymity, accessibility, adaptability, etc. These all serve to lower the threshold when seeking mental health support (Bauer et al., 2005; Mrazek et al., 2019). Simultaneously, new challenges and limitations have arisen in connection with the use of digital and/or online tools, e.g., confidentiality issues, low levels of engagement, or concerns regarding professionalism (Bauer et al., 2005). However, accessible, adaptable digital programs lower usage barriers in schools and other institutions, as they require relatively little expertise or effort compared to face-to-face (F2F) interventions. Web-delivered interventions may also improve fidelity by providing self-directed programs (Calear et al., 2016).

In addition to the high accessibility and availability of digital tools, their potential for successfully promoting young people’s mental health has repeatedly been reported in recent meta-analysis and/or systematic reviews. Harrer et al. (2019) found such tools to have positive effects on depression, anxiety, stress, eating disorder symptoms and role functioning. The findings of Clarke et al. (2015) and Sevilla-Llewellyn-Jones et al. (2018) support the effectivity of online interventions with respect to the treatment of anxiety and depressive symptoms. Noh and Kim (2022) reported beneficial results when preventing an increase in depressive symptoms, but not for anxiety or stress. Furthermore, well-tailored digital interventions are likely to increase engagement with a support tool and to aid the transfer of specific skills or strategies into the daily lives of young people (Lucas-Thompson et al., 2019). Indeed, web-based interventions have been reported to improve individuals’ quality of life and functioning (Sevilla-Llewellyn-Jones et al., 2018).

Extensive efforts have been made to provide systematic reviews on the issue of mental health provision. There have been reviews on older populations (Brown et al., 2016; Harrer et al., 2019; Noh & Kim, 2022), on clinical populations (Sevilla-Llewellyn-Jones et al., 2018), on F2F interventions (Carsley et al., 2018; Dray et al., 2017; Sapthiang et al., 2019), on school-based interventions (Cilar et al., 2020), but not on this specific focus, and in some cases the relevant meta-analysis has also been neglected (Clarke et al., 2015; Sapthiang et al., 2019). While previous reviews have reported on the efficacy of mobile apps (Bakker et al., 2016; Grist et al., 2017), the present review aims to include studies on mobile apps in addition to studies on other digital tools, thus expanding the range of intervention programs reviewed.

## Current study

Although existing reviews are of substantial scientific value, as the technological landscape is changing so rapidly an update on the effects of digitally-delivered interventions is clearly needed. In addition, the past systematic reviews had a different focus. The present systematic review and meta-analysis aims to outline the current state of digital, evidence-based programs promoting mental health in young people, and to provide insight into the characteristics and effectiveness of such programs. The domain of interest is the promotion of mental health as supported by digital technologies, with a focus on mental health literacy, well-being, (mental health) help-seeking behavior, stress management, relaxation, mindfulness, resilience and positive psychology. The present study focused on three areas. First, it was of interest to determine, what digital-based interventions promoting mental health are available for children and adolescents aged 11 to 18. Second, the effectiveness of these interventions was analyzed. Third, the factors underlying their effectiveness were assessed.

## Methods

This review makes use of the recommendations of the Preferred Reporting Items for Systematic Review and Meta-Analysis (PRISMA) statement (Page et al., 2021).

### Search Strategy

A comprehensive literature search was conducted (mid-May 2021) using the electronic databases PubMed, PsycInfo, and The Cochrane Library using the search strings stated in Table 1. A second search (end-October 2021) was run before the final analysis. Furthermore, registered trial protocols were checked for recently published studies and the reference lists of identified studies were searched manually in order to identify any potentially relevant literature.

**Table 1:** Search strings

PubMed and Cochrane	PsycINFO
(“Mental health” OR Wellbeing OR Well-being OR “Well being” OR e-health OR “life skills”) AND (Online OR Digital OR Mobile OR Phone OR App OR Textmessag* OR Web OR Computerized OR Computer-based) AND (“Help-seeking behavio*” OR Relaxation OR Stress OR Mindfulness OR Resilienc* OR “Positive psychology”) AND (Youth OR Adolescents OR students) AND (Training OR prevention OR Intervention OR Program NOT therapy)	(Mental-health OR Wellbeing OR Well-being OR e-health OR life-skills) AND (Online OR Digital OR Mobile OR Phone OR App OR Textmessag* OR Web OR Computerized OR Computer-based) AND (Help-seeking-behavio* OR Relaxation OR Stress OR Mindfulness OR Resilienc* OR Positive-psychology) AND (Youth OR Adolescents OR students) AND (Training OR prevention OR Intervention OR Program NOT therapy)

### Eligibility Criteria

Table 2 provides an overview of all criteria that determined whether a study was included.

**Table 2:** Exclusion and inclusion criteria of studies

	Inclusion criteria	Exclusion criteria
Participants	Children/adolescents aged between 11 to 18 years	Clinical sample
Intervention	Preventive interventions with ≥50% digital delivery	Clinical trials; therapy
Study type	Quantitative or mixed-methods studies	Qualitative studies
Study design	Controlled studies (CT) with pre-post comparison	No control group
Publication	Peer-reviewed; between 2000 and 2021	Prior to 2000
Language	English	Not English

Studies which focused on an age group outside the appointed range were included if the mean age of participants fell between 11 and 18 years. The WHO (World Health Organization) recommends that individuals between 10 and 19 years be regarded as adolescents (WHO, 1986). However, as the transition from childhood to adolescence and from adolescence to adulthood depends on several factors (genetic, nutritional, socioeconomic and demographic) and cannot occur at the same age for everyone, in practice one may need to be quite flexible (Canadian Paediatric Society, 2003). Despite this, in the present study it was decided on the narrower age span of 11–18 years, as 11 is the age where children in many countries transition out of primary school (e.g. Austria, UK, USA), and 18 is considered the legal age of majority in most countries around the world (Boyacıoğlu Doğru et al., 2018). The studies dealt with here focus on prevention rather than treatment, and target the general population, rather than participants with a self-reported or diagnosed mental illness.

The aim was to review interventions that promote mental health in general, and which target well-being, mental health literacy, resilience, help-seeking behavior, mindfulness, stress management, relaxation and positive psychology. Of particular importance was the application of a digital component, where the mode of delivery was either fully, or partly digitalized. Hence, interventions needed to be strongly supported by a digital component, with at least half the instructions being delivered in a digital format. However, whether the mode of delivery was online or offline (no internet connection necessary), was not relevant. Digital technologies and media continue to develop and evolve rapidly. To avoid the risk of basing the review on outdated or unavailable technologies, studies published before the year 2000 were not considered.

Studies with quantitative and mixed-methods were of interest, as quantitative data was needed to perform the planned meta-analysis. In order to be able to draw evidence-based conclusions, rigorous standards for quality and reliability of study results were indispensable. Thus, only peer-reviewed articles were included. Academic articles published in English were included. Considering that English is undoubtedly the dominant language in academic publications worldwide, and even journals in non-English-speaking countries increasingly favor English contributions (Murray & Dingwall, 2001), it was decided to focus on English publications.

### Study Selection Process

**Fig. 1:**
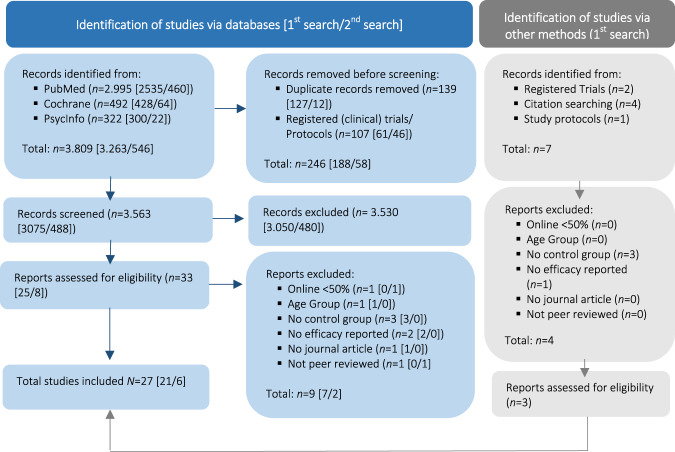
PRISMA flow chart (Page et al. 2021) of study selection process

In total, 27 studies were included in the sample. Figure 1 illustrates the steps of the study selection process and the reasons for exclusion, modeled according to PRISMA recommendations (Page et al., 2021).

Both searches, in May and in October, followed the same procedure. Four reviewers were involved in the study selection process and applied eligibility criteria for sample identification. As a first step, the literature search was conducted in all three databases and duplicates were removed. Secondly, all records were screened with respect to inclusion/exclusion criteria. Thirdly, doubtful cases for inclusion were double-checked and screened to ensure that they qualified for inclusion. Researchers were blinded to each other’s decisions in the first and second step only. Discrepancies and disagreements between individual judgements were resolved in online team meetings. If screening at the title and abstract level did not suffice, the full text was assessed by each reviewer in terms of the inclusion criteria. The software for recording decisions was Microsoft Excel, with more transparency being gained by using Google Sheets for the third step.

### Data Extraction

The Microsoft Excel software was used for duplication removal and screening of studies. To facilitate collaboration during the work process, the free online tool *SRDR*+*, Systematic Review Data Repository-Plus* (EPC, 2022) was used for data management. Data extraction included design details, arm details, sample characteristics, outcome details, and results. In a first step, all data extraction items were specified. Relevant extractions involved, but were not limited to: bibliographical data (e.g. author, publication year, country), theoretical background, medium(s) of intervention, mode of intervention, intervention characteristics, study design, method, sample characteristics, setting, outcome data/results, potential moderators of intervention efficacy, effect size and data recording acceptance and engagement. In a third step, findings were checked and discussed.

### Quality Assessment

In order to assess the risk of bias in the primary studies included, two independent reviewers applied assessment tools by Cochrane. For randomized controlled trials (RCT), the *Cochrane Risk of Bias Tool - RoB* (Higgins et al., 2022) was applied. For non-randomized trials the *Risk Of Bias In Non-Randomized Studies*–*ROBINS-I* (Sterne et al., 2016) was used. The tools help to detect the biases arising from pre-intervention, such as the bias in selection/randomization of participants, during intervention, such as the bias in classification of intervention, and post-intervention, such as the bias due to missing data (Sterne et al., 2016). The RoB tool allows for an overall bias rating of either low risk, some concerns, or high risk. For the ROBINS-I tool the overall rating scheme is more nuanced, and uses the categories low, moderate, serious and critical risk. Rating disagreements were resolved by calling on a third reviewer.

### Analyses

#### Narrative synthesis

In the process of narrative synthesis, the key features of the studies and their interventions were summarized, and potential intervention barriers and facilitators were put forward. In an extensive summary table, relevant study characteristics, such as the underlying theoretical framework, reported findings and outcome data were brought together. Additionally, a detailed description was provided for those extracted variables identified as potential moderators of intervention efficacy. All eight moderators were operationalized as categorical variables (Table 5). These were: level of interaction (none, some, considerable), level of professional support (none, some, considerable), level of guidance (none, some, considerable), level of digitization (fully digital, partly digital), duration of intervention (long [>3 months], medium [2-3 months], short [1 month], single session), level of adherence (consistent, inconsistent, not specified), level of attrition (no attrition, low [<20%], concerning [>20%]), and setting (homeschool, school, leisure, mixed).

#### Meta-analytic procedure

Meta-analyses were carried out using R (4.2.1 version), specifically using the meta package (Schwarzer, 2022) and the dmetar package (Harrer et al., 2019). In the event of missing data, the corresponding authors were contacted via email. In the case of no response, a friendly reminder was sent out 1 to 3 weeks later. Additional data sent by August 7, 2022, before performing the final analysis, were included.

As done in previous research (Harrer et al., 2019), conceptually-related clusters were created based on outcome frequency. The creation of clusters followed a strategic approach and entailed examining which mental health (MH) domain the respective scale was designed to measure. If a scale measured the same, or a closely related MH domain (e.g. stress and school stress), the items were combined in the same cluster. If studies used multiple measures for one construct, the measure with the highest Cronbach’s alpha or relevance was chosen (Supplementary Material 1). Studies that presented results for the same outcomes were combined to generate the average effect size of said outcome, regardless of the level of outcome scope (i.e., primary, secondary, tertiary, or explorative). If a study reported results applicable to more than one cluster it was included in all analyses for which it provided suitable outcomes. Clusters containing at least five studies were created and separate meta-analysis on each cluster were performed.

In line with common procedure the standardized mean difference (SMD) between the intervention groups and the control groups were calculated only for post-intervention measurements (Cuijpers et al., 2017; Harrer et al., 2019). Analyzing pre-post values was consciously rejected since estimating pre-post correlation within groups can lead to biased outcomes (Cuijpers et al., 2017). Adopting the guidelines suggested by Cochrane (Higgins et al. 2022), data from primary studies were arranged to ensure that their respective scales were in the same direction. To compensate for small sample sizes, the SMD was corrected, expressing it in the form of Hedges’ g (Hedges & Olkin, 2014). The effect size was interpreted in accordance with Cohen. Hence 0.2 is interpreted as a small, 0.5 as a medium, and 0.8 as a large effect. As significant differences between the studies were expected, it was decided to opt for a random effects pooling model, using the Hartung–Knapp–Sidik–Jonkman method for correction (IntHout et al., 2014; Sidik & Jonkman, 2022). The restricted maximum likelihood estimator (Viechtbauer, 2005) was used to calculate the heterogeneity variance τ^2^. The prediction interval (95%) was calculated around the size of the aggregated effect, thus showing the range of predictions required to reduce the actual effects of similar future trials based on existing evidence (Borenstein et al., 2017). Finally, the heterogeneity was assessed using the *I*^*2*^ statistic (Higgins & Thompson, 2002). Here Higgins et al. (2003) was adopted as a metric for interpretation: 25% low, 50% moderate, and 75% substantial heterogeneity (Higgins et al., 2003).

A sensitivity analysis was carried out when the heterogeneity between studies exceeded 50%. A first approach consisted in performing the analysis with no statistical outliers. In this case, outliers were controlled for by removing studies when the range of their 95% confidence interval (CI) was completely outside the size of the pooled effect. An additional influence analysis, *leave-one-out*, was carried out to assess the effect of individual studies on overall effects. This method consists of omitting one study at a time when calculating the effect size of the collection (Viechtbauer & Cheung, 2010). Further strategies were adopted to control for unit-of-analysis problems, such as multi-arm studies, where more interventions were compared to the same control conditions. These comparisons are not independent and may artificially reduce heterogeneity and distort the size of the combined effect (Borenstein et al., 2021). The solution adopted entailed combining the effects of all intervention groups into a single comparison and then recalculating the results (Higgins et al., 2022).

To study possible sources of heterogeneity, the results obtained from a sufficient number of studies (k > 10) were analyzed in subgroups. As a result, four clusters were included in the subgroup analysis: anxiety, depressive symptoms, internalizing symptoms, and protective factors. These clusters were analyzed against eight previously identified moderators: setting, interaction, support, guidance, digital, length/duration, adherence, and attrition.

Finally, to evaluate potential publication bias, funnel plots were inspected (Peters et al., 2008) and Egger’s test were performed (Egger et al., 1997) to assess funnel plot asymmetry. Where evidence of publication bias was found, the possible bias was adjusted using the Duval and Tweedie Trim and Fill procedure (Duval & Tweedie, 2000).

## Results

### Study Characteristics

The final sample included 27 studies. Eleven studies (41%) were implemented in Europe, 2 (7%) in Asia, 1 (4%) in Africa, 6 (22%) in North America and 7 (26%) in Australia. More than half of the studies (15/56%) reported significant intervention effects favoring the intervention group, with one-third being targeted (5/33%) and two-thirds being universal interventions (10/67%). For the rest, significant between-group intervention effects were not found. Three studies (11%) reported no between-group effects but did report significant within-group effects (#16, #25 and #26). Table 3 and the following narrative synthesis provide a summary of the studies’ key characteristics and specifically address the first research question–What digital-based interventions promoting mental health are available for children and adolescents aged 11 to 18?

**Table 3:** Study characteristics and main findings

#	Study (Country, Design)	Study sample (Mean age)	Conditions	*N*^a^	Theoretical background	Key intervention components	Setting Mode and method of delivery	Duration and frequency of intervention	Post-tests	Attrition rate(s)^b^	Main findings
1	Bohleber et al. (2016) (CHE, CT)	Employed apprentices (EA, 16.9) Unemployed adolescents (UA, 18.4) Total (17.7)	1. Companion App (EA) 2. Companion App (UA) 3. Control (EA) 4. Control (UA)	1.134	Positive peer culture	Peer mentoring app • Mentor = peer with same profession • Messaging and discussion groups • Counseling service & blog • Psychoeducation (Links to MH web content)	Leisure Online Mobile App	10 months Self-directed, NS	post-test Longi-tudinal data only analyzed for EA.	34.1% (EA: 54.6%)	No significant effects. Potential explanations may be the low frequency of app usage and the lack of need. However, concept of the app was well received.
2	Burckhardt et al. (2015) (AUS, RCT)	High school students (14.2)	1. Bite Back program 2. Control, alternate websites	336	Positive Psychology (PP)	Website with interactive activities and workbook • Gratitude entries • Mindfulness practices • Private and public postings • Psychoeducation on PP domains	School Partly online Website + Workbook	4–6 weeks 6 h total	post-test	40.9%	No significant effects (ITT). Delivery as a structured school program might explain the non-significant results (compare study #16). Both groups demonstrated improvements in depressive symptoms, stress and life satisfaction.
3	Calear et al. (2016) (AUS, Pilot RCT)	Secondary school students (15.0)	1. E-couch (worry & anxiety) program 2. Wait-list control	225	Cognitive behavior therapy (CBT) Mindfulness Progressive Muscle Relaxation (PMR)	Program with toolkits • CBT kit • Relaxation kit • Physical activity kit • Psychoeducation in each of the toolkits	School Online Web-based program	6 weeks 30–40 min/week	post-test 3 months	32.4% 43.1%	No significant effects (ITT). However, effect sizes of anxiety sensitivity (post-test, *d* = 0.19) and mental well-being (post-test, *d* = 0.17; FU: *d* = 0.30) were consistent with effects reported in other prevention trials.
4	Craig Rushing et al. (2021) (USA, RCT + crossover)	Adolescents identifying as AI/AN (range: 15–24)	1. BRAVE program 2. Control, STEM program	1.030	Principles of inclusion, equity belonging and diversity	Text messages with MH-related content • Resources, videos • Reflective questions • Q&A response • Psychoeducation	Leisure Online Mobile-based program	8 weeks 3 text messages/ week	3 months 5 months 8 months	8.4% 45.5% 50.1%	No significant effects. Both interventions improved measured health outcomes.
5	De la Barrera et al. (2021) (ESP, RCT)	High school students (12.6)	1. EmoTIC 2. Wait-list control	286	Ability model of emotional intelligence	Space adventure game and classroom sessions • Rebuild a spaceship • Group discussions • Reflections • Skill testing • Psychoeducation	School + leisure Partly online Game-based program + F2F sessions	4 weeks 1 classroom session/week, 3 home activities/ week	post-test	58.4%	Significant effects. BETWEEN: IG showed improved self-esteem (*η*2 = 0.12), affect balance (*η*2 = 0.06), emotional symptoms (*η*2 = 0.15), behavioral problems (*η*2 = 0.05) and hyperactivity (*η*2 = 0.06).
6	Douma et al. (2021) (NLD, RCT)	Adolescents with physical chronical illness (15.1)	1. Op Koers Online 2. Wait-list control	59	CBT	Group therapy sessions • Information seeking • Relaxation techniques • Social-competences • Positive thinking • Psychoeducation	Leisure Online Web-based program	8 weeks 90-min Life Chat/ week + booster session at 4 months	6 months 12 months	22% 13.6%	Significant effects (ITT). BETWEEN: Beneficial effects on disease-related coping skills (6-month FU: relaxation, *β* = 0.68; social competence, *β* = 0.57; info seeking, *β* = 0.52; 12-month FU: info seeking, *β* = 0.61) and health-related QoL (6-month FU: total HRQoL, *β* = 0.52; social-, *β* = 0.56; school-, *β* = 0.55; psychosocial-functioning, *β* = 0.60; 12-month FU: ns.).
7	Edridge et al. (2020) (GBR, c-RCT)	Primary and secondary school students (10.9)	1. ReZone 2. Control, school as usual	409	CBT Mindfulness Attention bias modification training (ABMT)	App for stress relief and refocus • Stress-related functions • Breathing exercise • Art therapy • Games	School + leisure Partly online Mobile App + F2F Tutorial	3 months 1-hour tutorial Self-directed, NS	post-test	16%	No significant effects (ITT). Potential reasons may have been implementation barriers of new digital interventions in schools and the lack of usage consistency.
8	Egan et al. (2021) (USA, RCT)	Adolescents identifying as SGMYs with cyberbullying experience (15.8)	1. Singularities 2. Wait-list control	240	Social cognitive theory Stress and coping theory Social and emotional learning framework	Role-playing game • Team setting to encourage help-seeking behavior • Use of productive coping skills • Psychoeducative web resources	Leisure Online Game-based program	4 months Self-directed, NS	1 month, 2 months	32.1% 35.8%	Significant effects (ITT). BETWEEN: IG reported lesser cyberbullying victimization, binge drinking, and marijuana use than CG. Interpret with caution: not powered for measuring efficacy (feasibility study).
9	Fridrici and Lohaus (2009) (DEU, CT)	Secondary school students (14.8)	1. Face-to-face, F2F 2. Online-school, OS 3. Online-home, OH 4. Control	904	Stress prevention	Stress prevention program with modules on: • Problem solving • Cognitive reconstruction • Support seeking • Relaxation • Time management	School + leisure Partly online Web-based + F2F program	8 weeks 90 min/week Online IGs: 2 F2F training sessions (baseline, 4 weeks)	post-test	NS	Significant effects. BETWEEN: Increase in knowledge gain in all 3 IGs (OS, *η*2 = 0.130; OH, *η*2 = 0.022; F2F, *η*2 = 0.060). Increase in positive thinking for OS (*η*2 = 0.021) and F2F (*η*2 = 0.041). Reduction of psychological stress in the F2F and OS condition (multivariate analysis was not significant).
10	Haug et al. (2021) (CHE, c-RCT)	Secondary and upper secondary school students (15.4)	1. SmartCoach 2. Control	1473	Social cognitive theory	Life-skills training program to prevent substance use • Text messages with quizzes, video and web links • Self-management • Social & substance resistance skills • Psychoeducation	Leisure Online Mobile-based program	22 weeks 2–4 texts/week, NS	6 months	16.3%	Significant effects (ITT). BETWEEN: IG reports lower amounts of alcohol consumed per month (*d* = −0.08), fewer cigarettes smoked per month (*d* = −0.13) and reduced perceived stress (*d* = −0.15).
11	Huppert and Johnson (2010) (GBR, CT)	Male students from fee-paying boy’s schools (14.5)	1. Mindfulness 2. Control, school as usual	155	Mindfulness-based stress reduction (MBSR)	Mindfulness training • Guided classroom sessions (teacher) • Guided home practice (CD)	School + leisure Partly digital CD- & video-based + F2F program	4 weeks F2F: 40 min/ week CD: 8 min/ day	post-test	13.6%	No significant effects. However, a significant positive association between the amount of individual practice and improvement in well-being and mindfulness was shown within the IG.
12	Kauer et al. (2012) (AUS, RCT)	Young people with mental health concerns (17.9)	1. Mobiletype + ESA 2. Control, Mobiletype	114	Emotional self-awareness (ESA) Stepped-care approach	Self-monitoring app to enhance ESA: • Mood • Current activity • Stress • Alcohol use • Cannabis use • Diet • Sleep • Exercise	Leisure Online Mobile-based program	2–4 weeks 4 monitoring prompts (1–3 min)/day	post-test 6 weeks	23.7% 24.5%	Significant effects (ITT). BETWEEN: Self-monitoring was shown to effectively decrease depressive symptoms in IG (*ĸ*² = 0.54), through the mediating effect of ESA (emotional self-awareness). Rumination decreased in both groups over time.
13	Kenny et al. (2020) (IRL, c-RCT)	Second-level school students (16.1)	1. CopeSmart 2. Control	560	Emotional self-awareness Positive coping strategies	App promoting self-management • Mood rating and mood history • Positive coping tips • Psychoeducation	School + leisure Online Mobile App	4 weeks Every day, self-directed, NS	post-test 8–10 weeks	9% 31%	No significant effects (ITT). A potential reason may have been the low levels of distress at baseline, which made improvements harder to detect.
14	Kutok et al. (2021) (USA, Pilot RCT)	Adolescents with past experience of cybervictimization (15.3)	1. IMPACT 2. Control, web-based resource packet	80	CBT Motivational interviewing (MI)	App and video session to prevent/reduce the effect of cyberbullying • CBT and MI-PowerPoint session • Daily mood rating with text messages response • On-demand mood messages	Leisure Online Video-conferencing tool + Mobile App	8 weeks Brief video intervention; 1 query + message/day	post-test 16 weeks	2.5% 8.3%	Significant effects (ITT). BETWEEN: IG had better overall well-being (post-test, *β* = 1.17; FU, *β* = 3.24), higher bystander self-efficacy (post-test, *β* = 2.65), decreased stress (post-test, *β* = -0.66; FU, *β* = -0.89), and higher perceived social support (FU, *β* = 3.50) than CG.
15	Malboeuf-Hurtubise et al. (2021) (CAN, Randomized cluster pilot study)	Elementary school students (11.3)	1. Mandala drawing 2. Emotion-based directed drawing	22	CBT Social-emotional learning Mindfulness	Art-based intervention • Mandala drawing (mindfulness-based) • Emotion-based drawing related to fear, worry and irritation	School Partly online Video-conferencing tool + analog drawing activity	5 weeks 45 min/week	post-test	0%	No significant effects. Both drawing interventions may be beneficial with regards to inattention and hyperactivity.
16	Manicavasagar et al. (2014) (AUS, RCT)	Young people (15.4)	1. Bite Back 2. Control, 2 youth-oriented control websites	235	Positive Psychology	Website with interactive activities • Methods for skill development in PP domains • Links to MH resources • Comments and discussions • Psychoeducation on PP domains	Leisure Online Website	6 weeks 1 h/week	post-test	34.5%	Significant effects. WITHIN: IG reported lower depressive symptoms and stress, and higher well-being scores. The most reduction was observed in depressive symptoms and stress scores for participants with high adherence. Interpret with caution: small groups - IG split into low/high adher.
17	O’Dea et al. (2020) (AUS, RCT)	Young people (14.82)	1. WeClick 2. Wait-list control	193	CBT Social learning Theory	Relationship-focused app • Interactive story-telling • Activities to overcome relationship difficulties and negative thinking • Problem solving • Help-seeking	Leisure Online Mobile App	Single-session 1 h	4-weeks 12 weeks	16.1% 40.4%	Significant effects (ITT). BETWEEN: Greater increase in well-being (*d* = 0.37) and help-seeking intentions (*d* = 0.36) for IG. Mental health outcomes can be improved with less than one hour of exposure with sustained effects at 12-week FU.
18	O’Dea et al. (2021) (AUS, c-RCT)	Secondary school students (14.3)	1. Smooth Sailing 2. Control, school as usual	1841	Theory of help-seeking for MH problems in youth Principles of stepped care	Service model to improve help-seeking intentions • Mood check-ins • Session with school counselor (if severe symptoms) • Psychoeducation: modules on general MH, anxiety, depression, and help-seeking	School Online Web-based stepped-care service model	12 weeks 5×10 min psycho-education 4 mood check-ins	6 weeks 12 weeks	24.8% 30.1%	Significant effects (ITT). BETWEEN: IG reported small improvements in help-seeking intentions (ES = 0.10) and a greater reduction in students who needed support for their mental health, but were not seeking help.
19	Osborn et al. (2020) (KEN, RCT)	Secondary school students (15.5)	1. Shamiri-Digital Wellness 2. Control, Study-skills	103	Growth mindset theory	Self-help intervention to reduce anxiety and depressive symptoms and promote well-being • Three modules: growth mindset, gratitude, and value/virtue affirmation • Psychoeducation within each module (learn, read, write)	School Digital program	Single-session ~ 1 h	2 weeks	0%	Significant effects (ITT). BETWEEN: IG experienced larger declines in depressive symptoms than CG (*d* = 0.50), even more substantial effects in the high-symptom subsample (*d* = 0.83). The anxiety reduction was steeper than that for depressive symptoms (*d* = 0.29), but the study-skills condition was also associated with a steep reduction in anxiety, preventing the difference from being significant.
20	Perkins et al. (2021) (GBR, RCT)	Students (16.6)	1. SSI 2. Control, school as usual	80	Growth mindset theory	Mindset intervention • Videos on coping • Multiple-Choice questions • Writing a letter of advice • Psychoeducation	School Online Web-based program	Single-session 30 min	post-test 4 weeks 8 weeks	2.5% 11.3% 47.5%	No significance testing (ITT; feasibility study). Favoring the IG: At 4-week FU: small ES for self-compassion, *g* = 0.41; self-esteem, *g* = 0.33; depressive symptoms & anxiety, *d* = −0.45; At 8-week FU: moderate ES for anxiety, *g* = −0.57 and small ES for self-esteem, *g* = 0.39, depressive symptoms & anxiety, *g* = −0.35.
21	Puolakanaho et al. (2019) (FIN, RCT)	Ninth grade students (15.3)	1. Youth COMPASS - iACT face group 2. Youth COMPASS - iACT group 3. Control	243	Acceptance and Commitment therapy	Program aiming to enhance psychological flexibility • Modules on acceptance, defusion, mindfulness, self-compassion, and adaptation skills • Motivational feedback and questions • Coaching sessions	School + leisure Online Web- and mobile-based program	5 weeks ≥ 6 exercises of 5–10 min/ week 1. iACT face: 45-min Coaching + weekly Whatsapping with coach 2. iACT: Weekly text messaging with coach	post-test	1.6%	No significant effects (ITT). Significant effects (PP). Reduction in symptoms of overall stress (*d* = 0.22) and an increase in academic buoyancy (*d* = 0.27) favoring the IGs. Gains were larger among those with higher stress levels.
22	Santor et al. (2007) (CAN, CT)	Junior and senior high school students (14.7)	1. Logon 2. No Logon	1775	Proactive health and wellness model Reactive health-needs model	Health magazine to promote MH literacy, early detection of difficulties & help-seeking • Information • Posting questions • Answers from MH professionals	Leisure Online Website	12 months self-directed	post-test	13.6%	Significant effects. BETWEEN: Small reduction in school worry. Positive correlation between frequency of web site use and actual help seeking behavior.
23	Schleider et al. (2020) (USA, RCT)	Female tenth grade students from rural, low-income high school (15.2)	1. Growing Minds 2. Control, Heart	222	Growth mindset theory	Program targeting growth mindsets: • Personality, self-regulation, and intelligence mindsets • Quizzes + feedback • Coping strategies • Writing exercise • Psychoeducation	School Online Web-based program	Single-session 45 min	post-test 4 months	0.5% 5%	Significant effects (ITT). BETWEEN: IG showed greater improvements in depressive symptom severity (*d* = 0.23) and larger reduction in their odds of reporting elevated depressive symptoms (*d* = 0.29).
24	Sousa et al. (2020) (PRT, CT)	Students (12.4)	1. F2F + TeenPower 2. F2F	353	Health Information Technology Acceptance Model (HITAM)	Mental health app • Resources (videos, infographics, tips) • Social support (discussions forums, chats, messages) • Self-monitoring (e.g. sleeping habits, physical activity) • Interactive training modules • Motivational tools	School + leisure Partly online F2F program + Web-based software and mobile app	6 months self-directed	post-test	42.4%	Significant effects. BETWEEN: Nutrition (*η*2 = 0.03), positive life perspective (*η*2 = 0.04) and global lifestyle (*η*2 = 0.02) increased in IG compared to CG. Older adolescents tended to show a significant increase in rates of stress management.
25	Van Vliet and Andrews (2009) (AUS, CT)	High school students (13.0)	1. Stress management 2. Control, de-facto	653	Stress management Coping strategies	Stress management program • Cartoon narrative • Coping strategies • Teaching of skills, e.g. breathing exercises, PMR • Psychoeducation on stress and coping	School Partly online Web-based program & activity book	Duration, NS 6 lessons 30 min/lesson	post-test 3 months	NS	Significant effects. WITHIN: Increase in knowledge (ES = 0.36), support-seeking coping (ES = 0.015), well-being (ES = 0.1). Decrease in avoidant coping (ES = 0.22), total difficulties (ES = 0.16), psychological distress (ES = 0.16).
26	Yuan (2021) (CHN, CT)	Middle school students (13.3)	1. Mindfulness training group 2. Control	180	Mindfulness training (MT)	Mindfulness training • Mindfulness trainings recordings • Daily checks if practice was completed	Homeschooling Online Web-based program	6 months 15 min of recordings/day	2 months 4 months 6 months	3.3%	Significant effects. WITHIN: Mindfulness training increased students’ resilience and emotional intelligence.
27	Zheng et al. (2021) (CHN, c-RCT)	Seventh grade students from secondary schools (13.5)	1. REAP + health session 2. Control, health session	954	20-20-20 rule	Peer-to-peer live streaming app • Workouts during recess • Live streaming or posting pictures of workout • Health information session	Homeschooling Online Mobile app + health training session	2 weeks 4×15 min/day 1 training session	post-test	6.1%	Significant effects (ITT). BETWEEN: Significant reduction in anxiety (*β* = −0.36) and eye strain (*β* = −0.15) in homeschooled children.

*AI/NA* American Indian/Alaska Native, *CG* control group, *CI* chronic illness, *c-RCT* cluster randomized controlled trial, *CT* controlled trial, *ESA* emotional self-awareness, *FU* follow-up(s), *F2F* face-to-face, *HRQoL* health-related quality of life, *IG* intervention group, *ITT* intention-to-treat analysis, *MH* mental health, *NS* none/not specified, *PPA* per-protocol analysis, *QoL* quality of life, *RCT* randomized controlled trial, *SGMY* sexual and gender minority youth, *STEM* science, technology, engineering, and mathematics

^a^N at baseline (allocated and/or randomized). Analyzed sample size of studies might differ from reported N in table

^b^Overall attrition rate, combined for IG and CG for each point of measurement

#### General study design details

**Fig. 2:**
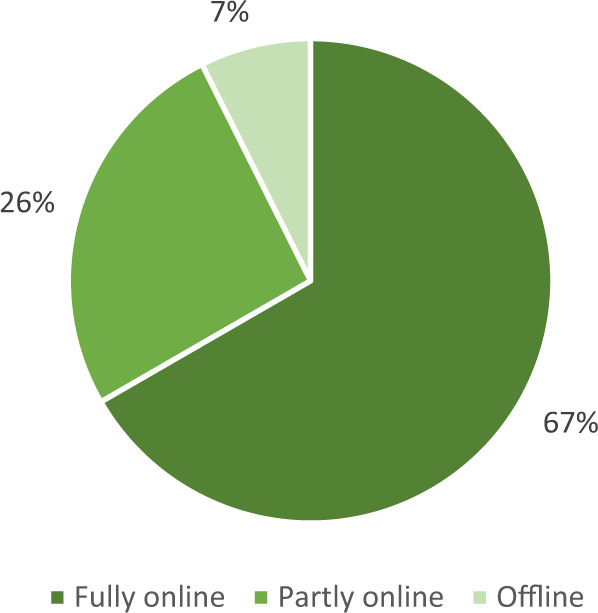
Mode of delivery of the MH interventions (*N* = 27)

**Fig. 3:**
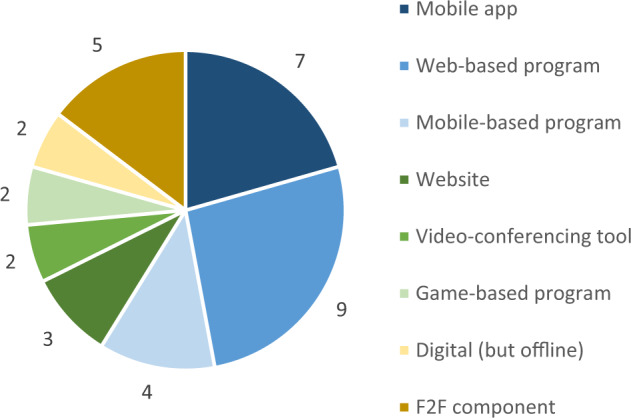
Media used to deliver the interventions. Note. Some interventions used multiple media

The RCT design was the one most commonly used (14/52%), about a fourth were cluster-RCTs (6/22%), and a little more than a fourth were CTs (7/26%). The common control group, where participants were not exposed to any intervention during the study period, was the preferred option (20/74%). About one quarter (7/26%) used an alternative program, that matched the intervention in duration and extent. In three of these cases (#4, #15, #19) both conditions led to improvements in outcome measures and non-significant between-group effects. For the great majority of studies (20/74%) intervention delivery was fully digital. The rest opted for partly digital delivery (7/26%). All but two studies delivered their interventions either fully (18/67%) or partly (7/26%) online (Fig. 2). Offline refers to a mode of delivery where no internet connection was necessary. The media used varied greatly, as shown in Fig. 3.

#### Participant characteristics

Participants’ mean age ranged from 10.9 to 17.9 (*M* = 14.65). Study #4 was ignored here as the authors only reported the age range (15–24 years). The sample sizes ranged from 22 to 1841 participants, giving a total of 13,857 participants at baseline, and 13,216 for final analyses. Studies #1, #22 and #25 did not report gender details. The remaining 24 studies (n = 9654) included 5313 (55%) females and 4176 (43%) males in their analyses. Only three of them (#4, #8, #14) reported on additional gender identification options (e.g., nonbinary or transgender). These made up 165 (2%) of all analyzed participants.

No significant moderating influence of gender on intervention effectiveness was found. A few gender differences were reported at baseline, i.e., higher initial overall stress and lower academic buoyancy (#21), higher stress vulnerability and more frequent use of social support (#9), more frequent and longer logons, as well as higher program engagement (#22) was reported for females. In contrast, more frequent use of avoidant coping (#9) was reported for male participants. Age affected intervention effectiveness in 4 (27%) of the studies that yielded significant results. On the one hand, younger adolescents were more likely to be absent at follow-up (#18) and showed a smaller improvement in coping with stress (#24) than older adolescents. On the other hand, younger participants were more likely to logon or post questions (#22), showed greater decreases in depressive symptoms, and showed greater improvements in happiness scores and improved mental well-being (#19) compared to older adolescents.

One study (#4) focused on youth identifying as American Indian and Alaskan native teenagers. Three studies (11%) focused on specific genders, namely females (#23), males (#11) and sexual gender minority youth (#8). Aside from gender, the most commonly measured sociodemographic characteristics were ethnicity (10/37%) and socio-economic status (9/33%). Neither was reported to have a moderating influence on the impact of the intervention. Participants were predominantly white and resided in the country where the intervention was implemented (as this was normally one of the inclusion criteria).

Easy access to, or possession of, a device (e.g., phone, tablet, computer) was mentioned by about one third (8/30%) as a criterion for inclusion. Not surprisingly, these were all non-school-based studies. Most studies opted for universal (22/81%), rather than targeted (5/19%) interventions. Studies with targeted interventions used (mental) health-related inclusion criteria, namely, low levels of resilience (#26), mild or more emotional mental health issues (#12), past-experiences with cyberbullying (#8, #14), and physical chronic illness diagnosis (#6).

Participants differed in their baseline levels of outcome measures. In line with expectations from previous research (Swain et al., 2015), participants with an elevated baseline in clinical symptoms and a low baseline in protective factors (e.g. resilience) seem to benefit more from an intervention. In the current sample this was true for elevated levels of depressive symptoms (#12, #19) and stress (#21), and low levels of resilience (#26). In contrast, study #5 found that adolescents with low and medium anxiety levels experienced an improvement in their self-esteem, whereas adolescents with high anxiety did not. However, in general, studies found that the higher the baseline scores (emotional intelligence, self-esteem, affect balance, and prosocial behavior), the lower the change found. Furthermore, some authors argued that the low levels of stress (#1), anxiety (#3), and distress (#13, #25), as reported by sample participants, were potential explanations of why no intervention efficacy was found.

#### Setting

The setting was classified in terms of homeschool, school, leisure-based and mixed ([home-]school- and leisure-based) as can be seen in Table 5. The studies comprised a balanced mix of school-based (10/37% [2/7% homeschooling]), leisure-based (10/37%) and mixed (7/26%) interventions.

### Risk of Bias

**Fig. 4:**
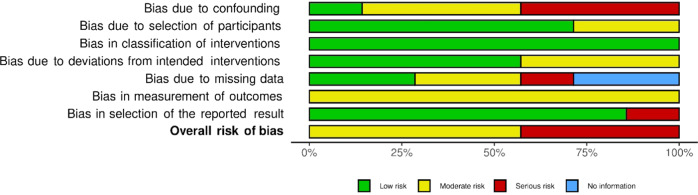
Summary plot ROBINS-I Assessment

**Fig. 5:**
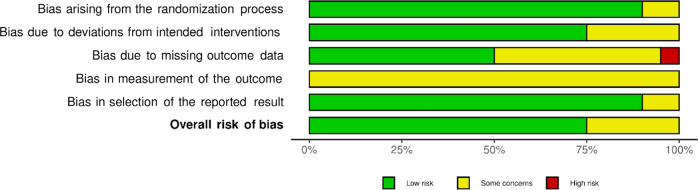
Summary plot RoB assessment

Of the 27 studies looked at, none of them was rated with a serious (RoB) or critical (ROBINS-I) risk of bias. Most of the randomized trials were rated as low risk (15/75%), one quarter raised some concerns (5/25%). For more than half (4/57%) of the non-randomized trials moderate concerns of bias were raised, whereas the remaining studies raised more serious concerns (3/43%). The present review reports on research that relied solely on self-reported measures and the blinding of participants was close to impossible, as is the case in psychotherapy research in general (Edridge et al., 2020). Therefore, the risks regarding such criteria are not considered as strictly as would be the case for clinical or medical trials. Figures 4 and 5 depict summary plots created with Robvis (McGuinness & Higgins, 2021), and provide more detail on judgement percentages.

### Intervention Characteristics

#### Availability

The availability of the interventions was assessed based on their online accessibility. Live sessions were used by three studies (11%; #6, #11, #15). These intervention programs are not available online. About half of the programs (13/48%) are available for students, either open access and for free (9/69%), or through an institution (4/31%), e.g. a school, for free (2/15.5%), or for a fee (2/15.5%). Of the remaining studies (11/41%), the reported weblinks resulted in error messages (3/27%), or no links were reported and access to the programs could not be found (8/73%; (Supplementary Material 2).

#### Domains and measures

As described above, defining mental health is quite challenging, and this is mirrored in the diversity of the attempts at its promotion seen in the current sample. Only one study (#9) focused promotional effort on a single MH domain, i.e., on stress. All others targeted multiple areas of MH. Among these, some (8/30%) specifically reported focusing on two domains, while others (18/70%) aimed at promoting mental health in a more general sense. This was reflected in the heterogeneity and quantity of the outcome measures applied. The outcomes most frequently measured were anxiety (11/41%), depressive symptoms (10/37%), internalizing symptoms (8/30%), well-being (7/26%), stress (6/22%), and help-seeking behavior (6/22%). Externalizing symptoms (5/19%), resilience (3/11%), mindfulness (2/7%), or intrapersonal factors, such as self-efficacy (4/15%) or self-esteem (3/11%), were measured less often. Similarly, a relatively broad spectrum of activities was also found. Psychoeducative elements (e.g. MH definitions, descriptions, symptoms, treatment options, information on MH domains, links to MH web content or external resources) were by far the most common items incorporated in the interventions (23/85%), followed by elements that encourage help-seeking behavior (13/48%), mindfulness practices (8/30%), reflective questions and problem solving (8/30%), possibilities of peer exchange (8/30%), coping skills training (7/26%), and mood ratings or check-ins (5/19%). The most frequently used theoretical frameworks were cognitive behavioral therapy (CBT, 6/22%), mindfulness (5/19%), growth mindset theory (3/11%), and social(-emotional) learning theory (3/11%). Since intervention length, personal interaction, professional support and guidance are particularly relevant with respect to intervention impact, these indicators were looked at more closely.

#### Intervention length

Exposure to the intervention varied widely across studies. There were interventions with only one session (4/15%), interventions that included multiple sessions (17/63%), or interventions with no specified sessions (6/22%). The majority of the studies (14/54%) used an intervention period of 2 to 8 weeks. Four studies (15%) used single-session interventions (approx. 60 min) and one (#25) did not specify its duration. The remaining third (8/30%) reported longer intervention times ranging between 12 and 48 weeks. In summary, the studies analyzed and presented the duration of the intervention differently, with some not having structured sessions. This variability was accounted for by operationalizing and classifying intervention periods into long [>3 months], medium [2-3 months], short [1 month], and single session interventions.

#### Level of interaction

The level of interaction refers to the nature of received feedback and to the opportunities for participant interaction within the intervention. Only a small minority (3/11%) of the described interventions failed to use any means of engagement (level 0). Some form of interaction, such as peer-feedback, automated responses, and/or little (or no) exchange with professionals (level 1), was used by more than half (15/56%). A considerable level of interaction (level 2), meaning the intervention involved adaptive, individually-tailored, computerized responses, or considerable interaction with peers or professionals, was exhibited by one third (9/33%).

#### Level of professional support

The level of support relates to the involvement of a professional in the intervention. One quarter (7/26%) of the studies made no mention of professional support (level 0). Some level of support by a professional, e.g. a teacher or research assistant, or supervision by a mental health professional was integrated into almost half the studies (13/48%, level 1). The remaining quarter (7/26%) reported on the use of more substantial support, whereby a mental health professional was present or actively involved throughout the intervention (level 2).

#### Level of guidance

The level of guidance refers to the amount of structure and direction given on how and when to practice or use a certain tool/program. Relatively few studies (3/11%) provided no structure or guidance (level 0). Half of them (14/52%) used interventions that were mostly self-directed, and where only limited prompts or reminders were employed so as to ensure participants’ adherence (level 1). The designation *considerable guidance* was used when more than 50% of the protocol was guided, modules and activities followed a set structure, or the intervention was supervised at fixed times within a certain institution, such as a school (level 2). This was found to be the case for little over a third of the studies (10/37%).

#### Level of digitization

**Fig. 6:**
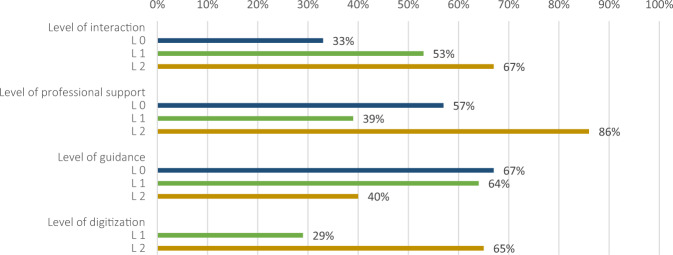
Percentage of studies that reported significant effects (*n* = 15) listed with their respective levels of interaction, professional support, guidance and digitization

Regarding the level of digitization, it was distinguished between level 1 interventions, which had a F2F component and were partly digital (7/26%), and level 2 interventions that were fully digital (20/74%). A level 0 was not defined, as some level of digitization was a criterion for inclusion in the sample. Figure 6 demonstrates how the levels of interaction, professional support, guidance and digitization coincided with significant intervention outcomes. As expected, a more intense level of interaction and professional support is associated with larger intervention effects. Also, fully digital interventions reported larger effects. The results regarding the subgroup analysis are reported below (Table 5).

### Adherence and Attrition

Attrition refers to participant dropout. Adherence pertains to user engagement, in other words, how well the participants complied with the intervention protocol. Both adherence and attrition are major concerns in mental health intervention studies (Sousa et al., 2020). Higher engagement often leads to higher effectivity. This has been reported in prior research and is also found in the current sample (studies #2, #11). The reporting on adherence differed greatly within the studies, and some studies (5/19%) did not predefine an adherence criterion at all. However, it was distinguished between *rather consistent* and *rather inconsistent* levels of adherence. Rather inconsistent engagement was attributed to studies where participants completed less than 50% of tasks/days/modules/activities or where app/program usage was reported to be low by the authors themselves. A little less than half (13/48%) of the studies reported consistent levels of adherence, one third (9/33%) inconsistent levels, and four (15%) did not report on adherence at all. Study #19 reported different adherence levels for its intervention groups, with inconsistent adherence for the leisure-based group and consistent adherence for the school-based group.

Closely related to the concept of adherence is that of attrition. The level of attrition relates to the overall dropout rate of participants from baseline to post-test or follow-ups. A dropout of 20% or less was considered to be low attrition (level 1), a dropout of 21% and above, was designated as *concerning* attrition (level 2). The cut-offs were chosen in accordance with previous findings, which suggest a concern for bias is called for when attrition rates exceed 20% (Marcellus, 2004). In the current sample, the overall attrition rates ranged between 0 and 58.4% (*M* = 25.2%) and were self-computed in most cases due to a lack of specific reporting. Two studies (#9, #25) could not be taken into account due to insufficient data, and two reported zero attrition (#15, #19). Generally speaking, attrition rates increased across time points (*M*_T2_ = 19.7%; *M*_T3_ = 26.2%; *M*_T4_ = 33.6%). At post-test (n_T2_ = 25) low levels of attrition were found for more than half the studies (15/60%), concerning levels for the rest (10/40%). At follow-up (n_T3_ = 11) low levels were found for about a third (4/36%), and concerning levels for almost two thirds (7/64%). For studies with a second follow-up (n_T4_ = 3), 2 out of 3 (67%) showed concerning levels.

### Meta- and Sensitivity Analysis

Separate analyses for each cluster were conducted in order to address the second research question on how effective these interventions are. As mentioned above, seven clusters were created: anxiety, depressive symptoms, externalizing symptoms, internalizing symptoms, protective factors, stress, and well-being (Supplementary Material 1). The outcome measures in the clusters well-being, anxiety, depressive symptoms and stress were largely homogenous, this means they specifically measured the MH domain in question. The other clusters consisted of more heterogenous measures that were combined due to their conceptual relatedness. The cluster internalizing symptoms included measures of emotional symptoms, internalizing behavioral problems or rumination. The cluster externalizing symptoms contained hyperactivity, behavioral problems or difficulties. Lastly, the cluster protective factors contained measures of self-esteem, self-efficacy or help-seeking behavior.

**Fig. 7:**
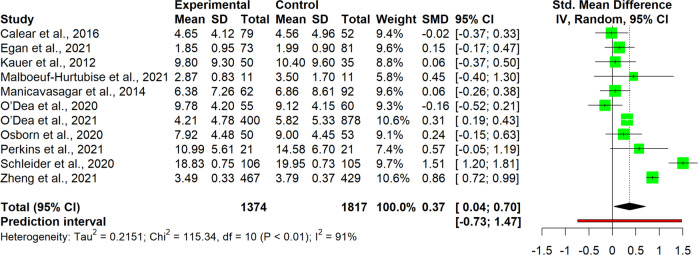
Forest plot for anxiety outcomes

**Fig. 8:**
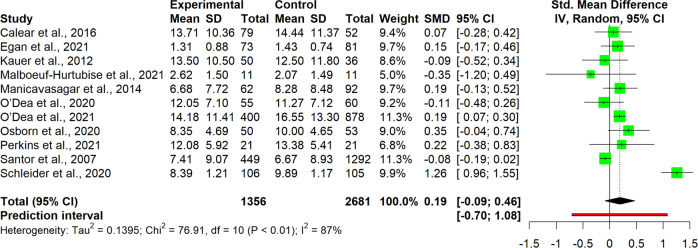
Forest plot for depressive symptoms outcomes

**Fig. 9:**
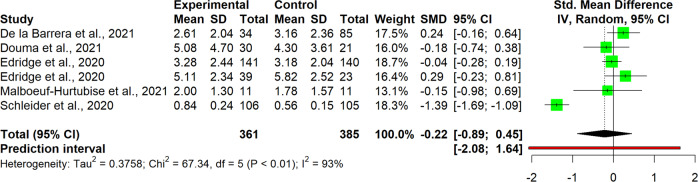
Forest plot for externalizing symptoms outcomes

**Fig. 10:**
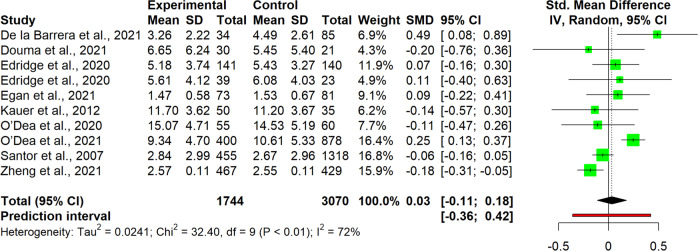
Forest plot for internalizing symptoms outcomes

**Fig. 11:**
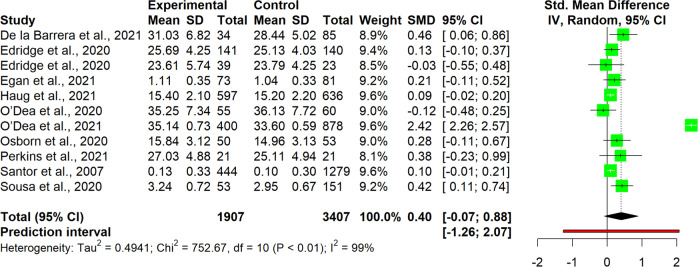
Forest plot for protective factors outcomes

**Fig. 12:**
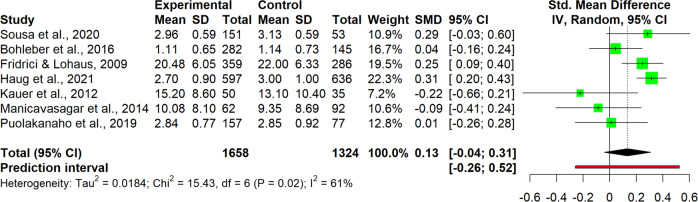
Forest plot for stress outcomes

**Fig. 13:**
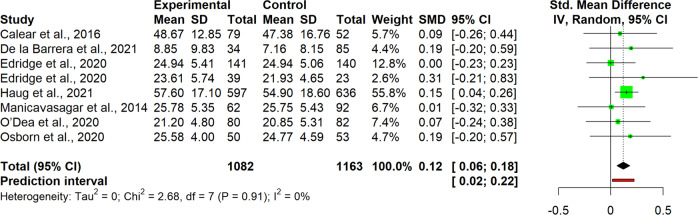
Forest plot for well-being outcomes

The interpretation of results was carried out for single clusters. This entailed analyzing the observed pooled effects through forest plots (Figs. 7–13), the potential publication bias as depicted by asymmetry in funnel plots (Supplementary Material 3) and detected by Egger’s test (Supplementary Material 4), and looking at the heterogeneity and relative strategies adopted to correct for this in the sensitivity analysis (Table 4 and Supplementary Material 5). Additional data was needed from 15 studies, so their respective authors were contacted. Data was provided by studies #1, #5, #7, #8, #12 and #18. As no additional information was obtained from the remaining nine studies (with either no data [#9, #14, #25], or no response [#2, #4, #13, #21, #22, #26]), they were not part of the following considerations.

**Table 4:** Pooled effects per cluster and sensitivity analysis

Cluster		Effect size			Heterogeneity			
	*n*	*g*	*95% CI*	*p*	*I*^*2*^	*95% CI*	*p*	*95% PI*
Anxiety	11	0.37	[0.04, 0.70]	0.031	91	[87, 94]	<0.001	[−0.73, 1.47]
Outliers removed^a^	9	0.16	[0.02, 0.31]	0.033	29	[0, 67]	0.183	[−0.15, 0.48]
Influence analysis^b^	10	0.25	[−0.02, 0.48]	0.034	87	[78, 92]	<0.001	[−0.46, 0.97]
Depressive symptoms	11	0.19	[−0.09, 0.46]	0.163	87	[79, 92]	<0.001	[−0.71, 1.08]
Outliers removed^b^	10	0.07	[−0.05, 0.19]	0.198	46	[0, 74]	0.054	[−0.21, 0.36]
Influence analysis^b^	10	0.07	[−0.05, 0.19]	0.198	46	[0, 74]	0.054	[−0.21, 0.36]
Externalizing symptoms	6	−0.22	[−0.89, 0.45]	0.434	93	[87, 96]	<0.001	[−2.08, 1.64]
Outliers removed^b^	5	0.03	[−0.19, 0.25]	0.719	0	[0, 79]	0.535	[−0.25, 0.31]
Influence analysis^b^	5	0.03	[−0.19, 0.25]	0.719	0	[0, 79]	0.535	[−0.25, 0.31]
Internalizing symptoms	10	0.03	[−0.11, 0.18]	0.624	72	[47, 85]	<0.001	[−0.36, 0.42]
Outliers removed	–	–	–	–	–	–	–	–
Influence analysis^c^	9	−0.02	[−0.16, 0.12]	0.714	40	[0, 73]	0.099	[−0.31, 0.27]
Protective factors	11	0.40	[−0.07, 0.88]	0.089	99	[98, 99]	<0.001	[−1.26, 2.07]
Outliers removed^c^	10	0.13	[0.05, 0.21]	0.006	12	[0, 53]	0.332	[0.05, 0.20]
Influence analysis^c^	10	0.13	[0.05, 0.21]	0.006	12	[0, 53]	0.332	[0.05, 0.20]
Stress	7	0.13	[−0.04, 0.31]	0.107	61	[11, 83]	0,017	[−0.26, 0.52]
Outliers removed	–	–	–	–	–	–	–	–
Influence analysis^d^	6	0.09	[−0.09, 0.27]	0.266	42	[0, 77]	0.122	[−0.27, 0.44]
Well-being	8	0.12	[0.06, 0.18]	0.003	0	[0, 68]	0.913	[0.02, 0.22]
Outliers removed	–	–	–	–	–	–	–	–
Influence analysis	–	–	–	–	–	–	–	–

^a^Removed: “Schleider et al. (2020)”, “Zheng et al. (2021)”

^b^Removed: “Schleider et al. (2020)”

^c^Removed: “O’Dea et al. (2021)”

^d^Removed: “Haug et al. (2021)”

#### Anxiety

For studies of the anxiety cluster (*n* = 11), the pooled effect size for intervention was found to be significant at a small-to-medium level, with *Hedges’ g* at 0.37, 95% CI [0.04, 0.70], *p* = 0.031 (Fig. 7). High heterogeneity was detected, with a significant *I*^*2*^ of 91% (95%CI [87, 94], *p* < 0.001), confirmed also through the quite wide prediction interval (95% PI [−0.73, 1.47]), thus indicating that negative intervention effects cannot be ruled out for future studies. Since heterogeneity was substantial, it was deemed necessary to proceeded with a sensitivity analysis. The influence analysis pointed out one study as the most influential (#23), and one that increased heterogeneity (#27), which also happened to be an outlier. After removing outliers and re-running the analysis, the level of heterogeneity fell and was no longer significant (*I*^*2*^ = 29%, 95% CI [0, 67], *p* = 0.183), while the pooled effect size decreased but remained significant, *g* = 0.16, 95% CI [0.02, 0.31]. No evidence for publication bias was found, neither in the funnel plots, nor after performing the Egger’s test.

#### Depressive symptoms

Studies of the depressive symptoms cluster (*n* = 11) revealed a small and non-significant effect size for intervention, *g* = 0.19, 95% CI [−0.09, 0.46] (Fig. 8). High heterogeneity was detected (*I*^*2*^ = 87%, 95% CI [79, 92]), *p* < 0.001), carrying a high variability in predicting further results (95% PI [−0.71, 1.08]). Both approaches adopted in the sensitivity analysis pointed in the same direction, identifying the same highly influential/outlier study as in the anxiety cluster (#23). Omitting this study reduced the heterogeneity (*I*^*2*^ = 46%, 95% CI [0, 74], *p* = 0.054), at the expense of an even thinner and non-significant effect size for intervention, *g* = 0.07, 95% CI [−0.05, 0.19]. Funnel plots and Egger’s test found no indication for publication bias.

#### Externalizing symptoms

The cluster externalizing symptoms included only six studies. There is thus a need for caution regarding its interpretation. Results seem to buck the trend of other analyses, since here the effect size favors the control rather than the intervention group, although there is no statistical significance, *g* = −0.22, 95% CI [−0.89, 0.45] (Fig. 9). High heterogeneity was also found within this cluster, *I*^*2*^ = 93%, 95% CI [87, 96], 95% PI [2.08, 1.64], *p* < 0.001. According to the sensitivity analysis, study #23 was again detected as an outlier. Its removal brought the effect size to positive values although they still remained non-significant, *g* = 0.03, 95% CI [−0.89, 0.45]. Surprisingly enough, the study removed accounted for all the heterogeneity in the cluster, since after its omission, the *I*^*2*^ was zero and non-significant, 95% CI [87, 96], *p* = 0.535. No signs of publication bias were detected in the Egger’s test.

#### Internalizing symptoms

Studies of the internalizing symptoms cluster (*n* = 10) revealed a small and non-significant pooled effect size, *g* = 0.03, 95% CI [−0.11, 0.18] (Fig. 10). Heterogeneity between studies was high and significant (*I*^*2*^ = 72%, 95% CI [47, 85], *p* < 0.001), although the prediction interval was not as extreme as in other cases, 95% PI [−0.36, 0.42]. In the sensitivity analysis, no outliers were detected. However, one influential study (#18) was omitted in the influence analysis, lowering the heterogeneity to an *I*^*2*^ of 40%, 95% CI [0, 73], *p* = 0.099. As a consequence, the pooled effect size remained small and non-significant, although changing sign, *g* = −0.02, 95% CI [−0.16, 0.12]. Egger’s test revealed no indication for publication bias.

#### Protective factors

Studies in the protective factors cluster (*n* = 11), showed a medium-sized but non-significant pooled effect, *g* = 0.40, 95% CI [−0.07, 0.88] (Fig. 11). Heterogeneity was extremely high and significant (*I*^*2*^ = 99%, 95% CI [98, 99], *p* < 0.001), providing low predictive power for further research, 95% PI [−1.26, 2.07]. Both strategies of adjustment adopted in the sensitivity analysis pointed to the same study (#18), both as an outlier, and as well as being the most influential. Removal of this study reduced the heterogeneity significantly to an *I*^*2*^ of 12%, 95% CI [0, 53]. Interestingly enough, after removing the study the pooled effect size was also affected significantly. While it became smaller, it also became significant, *g* = 0.13, 95% CI [0.05, 0.21], *p* = 0.006. Egger’s test revealed no indication for publication bias.

#### Stress

As the stress cluster contained only 7 studies, interpretation of the results requires considerable caution. The pooled effect size was small and non-significant, *g* = 0.13, 95% CI [0.04, 0.31] (Fig. 12). Heterogeneity was present, but moderate-to-large with an *I*^*2*^ of 61% (95% CI [11, 83], 95% PI [−0.26, 0.52]), so sensitivity analysis was also conducted in this case. While no outliers were detected, omitting one particular study (#10) led to a strong reduction in heterogeneity, resulting in an *I*^*2*^ of 42%, 95% CI [0, 77]. There were no big differences in effect size, which remained small and non-significant, *g* = 0.09, 95% CI [−0.09, 0.27]. This was the only cluster where the Egger’s test revealed a risk for publication bias, with intercept = −2.89, 95% CI [−4.69, −1.10], *p* = 0.025. As a strategy for adjustment, the Duval and Tweedie Trim and Fill procedure was applied, which imputed two additional studies to adjust for publication bias. As a result, the effect size improved slightly and became significant, *g* = 0.20, 95% CI [0.00, 0.40], *p* = 0.049.

#### Well-being

Studies in the well-being cluster (*n* = 8) revealed a small but significant pooled effect size for interventions, *g* = 0.12, 95% CI [0.06, 0.18], *p* = 0.003 (Fig. 13). No heterogeneity was detected among the included studies (95% CI [0, 68]) and the prediction interval was quite narrow, 95% PI [0.02, 0.22]. Apparently, in this case, rather than using a random effects model, a fixed one would have been more appropriate. Egger’s test revealed no indication for publication bias.

### Subgroup Analysis: Facilitators and Barriers

In order to answer the third research question–*What are the factors underlying their effectiveness?*–eight moderators were identified and included in the subgroup analysis. These were: level of interaction, level of professional support, level of guidance, level of digitization, duration of intervention, level of adherence, level of attrition, and setting. With regard to the subgroup/moderator analysis, only significant results were reported here. Should more detail be required, the reader is referred to Table 5.

**Table 5:** Subgroup analysis for selected clusters

Group	Anxiety					Depressive symptoms					Internalizing symptoms					Protective factors				
	*n*	*g*	*95% CI*	*I*^*2*^	*p*	*n*	*g*	*95% CI*	*I*^*2*^	*p*	*n*	*g*	*95% CI*	*I*^*2*^	*p*	*n*	*g*	*95% CI*	*I*^*2*^	*p*
Setting					<0.001					0.109					<0.001					0.130
Homeschool	1	0.86	[0.72, 0.99]	–		–	–	–	–		1	−0.18	[−0.31, −0.05]	–		–	–	–	–	
Leisure	4	0.04	[−0.17, 0.25]	0		5	−0.02	[−0.18, 0.14]	4		5	−0.06	[−0.13, 0.02]	0		4	0.09	[0.00, 0.18]	0	
Mixed	–	–	–	–		–	–	–	–		3	0.20	[−0.36, 0.76]	36		4	0.26	[−0.08, 0.59]	30	
School	6	0.51	[−0.07, 1.09]	91		6	0.34	[−0.22, 0.89]	89		1	0.25	[0.13, 0.37]	–		3	1.05	[−1.97, 4.06]	99	
Interaction					0.622					0.777					0.420					0.731
Considerable	2	0.19	[−1.07, 1.45]	0		2	0.06	[−2.41, 2.52]	16		3	0.16	[−0.65, 0.96]	53		4	0.24	[−0.04, 0.53]	52	
None	3	0.19	[−0.47, 0.84]	30		3	0.20	[−0.20, 0.59]	0		–	–	–	–		2	0.31	[−0.29, 0.91]	0	
Some	6	0.45	[−0.21, 1.11]	95		6	0.23	[−0.32, 0.78]	93		7	0.00	[−0.16, 0.15]	77		5	0.51	[−0.83, 1.85]	99	
Support					0.019					0.739					0.960					0.305
Considerable	1	0.31	[0.19, 0.43]	-		2	0.05	[−1.65, 1.76]	91		3	0.05	[−0.48, 0.58]	87		3	0.98	[−2.14, 4.10]	99	
None	4	0.07	[−0., 0.33]	0		4	0.14	[−0.14, 0.42]	0		2	0.01	[−1.27, 1.28]	0		4	0.10	[−0.05, 0.25]	0	
Some	6	0.59	[−0.02, 1.20]	90		5	0.27	[−0.52, 1.05]	91		5	0.04	[−0.29, 0.36]	67		4	0.20	[−0.10, 0.51]	2	
Guidance					0.433					0.308					0.694					<0.001
Considerable	5	0.56	[−0.20, 1.32]	92		5	0.36	[−0.37, 1.10]	89		1	−0.20	[−0.76, 0.36]	-		2	0.31	[−0.29, 0.91]	0	
None	1	0.15	[−0.17, 0.47]	–		2	−0.02	[−1.33, 1.30]	45		4	−0.01	[−0.13, 0.11]	0		4	0.11	[0.03, 0.18]	0	
Some	5	0.25	[−0.24, 0.75]	93		4	0.12	[−0.10, 0.34]	15		5	0.06	[−0.29, 0.41]	86		5	0.66	[−0.60, 1.93]	99	
Digital					0.847					0.205					0.133					0.509
Fully digital	10	0.36	[0.00, 0.73]	92		10	0.22	[−0.07, 0.50]	88		7	−0.02	[−0.18, 0.14]	78		7	0.49	[−0.33, 1.30]	99	
Partly digital	1	0.45	[−0.40, 1.30]	–		1	−0.35	[−1.20, 0.49]	–		3	0.20	[−0.36, 0.76]	36		4	0.26	[−0.08, 0.59]	30	
Length					0.673					0.180					0.147					0.325
Long (>3 mo)	1	0.15	[−0.17, 0.47]	–		2	−0.02	[−1.33, 1.30]	45		2	−0.04	[−0.63, 0.54]	0		4	0.12	[−0.02, 0.26]	29	
Mid (2-3 mo)	4	0.19	[−0.10, 0.48]	38		4	0.17	[0.03, 0.30]	0		4	0.15	[−0.08, 0.38]	22		3	0.85	[−2.56, 4.26]	99	
Short (1 mo)	2	0.49	[−4.54, 5.52]	92		1	−0.09	[−0.52, 0.34]	-		3	0.03	[−0.88, 0.94]	79		1	0.46	[0.06, 0.86]	–	
Single session	4	0.55	[−0.61, 1.70]	94		4	0.45	[−0.50, 1.40]	92		1	−0.11	[−0.47, 0.26]	-		3	0.14	[−0.52, 0.79]	32	
Adherence					0.950					<0.001					0.547					0.599
Consistent	6	0.40	[−0.24, 1.03]	92		6	0.31	[−0.23, 0.85]	90		4	−0.05	[−0.26, 0.16]	0		4	0.15	[−0.17, 0.48]	4	
Inconsistent	4	0.32	[−0.31, 0.96]	94		3	0.18	[0.07, 0.28]	0		4	0.05	[−0.26, 0.37]	87		5	0.62	[−0.66, 1.89]	99	
ns	1	0.45	[−0.40, 1.30]	-		2	−0.09	[−0.52, 0.35]	0		2	0.18	[−3.25, 3.61]	85		2	0.23	[−1.98, 2.43]	66	
Attrition					0.007					0.803					0.051					0.227
Concern (>20%)	7	0.14	[−0.04, 0.33]	46		7	0.14	[0.05, 0.24]	0		5	0.14	[−0.14, 0.43]	50		6	0.64	[−0.32, 1.60]	98	
Low (<20%)	2	1.17	[−2.96, 5.30]	93		2	0.58	[−7.92, 9.08]	99		5	−0.08	[−0.21, 0.06]	16		4	0.10	[0.06, 0.14]	0	
No attrition	2	0.28	[−0.75, 1.30]	0		2	0.10	[−4.18, 4.38]	55		–	–	–	–		1	0.28	[−0.11, 0.67]	–	

The setting moderated the effects for two clusters, anxiety (*p* < 0.001) and internalizing symptoms (*p* < 0.001). Effects were significantly higher when the setting was at school, *g*_*anxiety*_ = 0.51, 95% CI [−0.07, 1.09]; *g*_*internalizing*_ = 0.25, 95% CI [0.13, 0.37]. Effects were the lowest in both clusters when the intervention was leisure-based, *g*_*anxiety*_ = 0.04, 95% CI [−0.17, 0.25]; *g*_*internalizing*_ = −0.06, 95% CI [−0.13, 0.02]. Further results indicated that for anxiety a homeschooling setting carried an even higher effect size (*g* = 0.86, 95% CI [0.72, 0.99]), although there was only one study included in this category. Finally, for internalizing symptoms a mixed setting generated a modest effect size for *Hedges’ g* at 0.20, 95% CI [−0.36, 0.76].

As for professional support, moderating effects were found within the anxiety cluster. Effects were significantly higher for studies that reported some level of support in their intervention (*g* = 0.59, 95% CI [−0.02, 1.20]), when compared to studies providing considerable support (*g* = 0.31, 95% CI [0.19, 0.43]), and to studies where no support was given, *g* = 0.07, 95% CI [−0.18, 0.33], *p* = 0.019.

With regard to the level of guidance, effects were found to be significantly higher within the protective factors cluster when the intervention was administered with some guidance (*g* = 0.66, 95% CI [−0.60, 1.93]), compared to when considerable (*g* = 0.31, 95% CI [−0.29, 0.91]) or no guidance (*g* = 0.11, 95% CI [0.03, 0.18], *p* < 0.001) was administered.

The level of adherence significantly moderated the effects for the depressive symptoms cluster (*p* < 0.001). Here, studies with consistent adherence showed greater effect size (*g* = 0.31, 95% CI [−0.23, 0.85]) compared to studies indicating inconsistent adherence (*g* = 0.18, 95% CI [0.07, 0.28]), and also to studies which did not indicate the level of adherence (*g* = −0.09, 95% CI [−0.52, 0.35].

Lastly, the level of attrition also was also shown to significantly moderate effect size in the anxiety cluster, *p* = 0.007. Studies with a low level (<20%) of attrition revealed a greater effect size (*g* = 1.17, 95% CI [−2.96, 5.30]) compared to studies that showed a concerning level of attrition (*g* = 0.14, 95% [−0.04, 0.33]), or to studies showing no attrition at all, *g* = 0.28, 95% CI [−0.75, 1.30].

## Discussion

Digital tools are increasingly being used to try to counteract the declining mental health of adolescents (Bergin et al., 2020). The range and variety of these tools is growing rapidly, and more and more studies report on their potential and value (Lucas-Thompson et al., 2019; Sommers-Spijkerman et al., 2021). An updated overview of tools and programs is essential. Therefore, this systematic review examined digital and/or online evidence-based prevention programs for the promotion of mental health in young people aged 11 to 18 years old. In total 27 studies were identified to meet the inclusion criteria. Half of these studies reported significant effects in improving mental health. A meta-analysis was performed based on post-intervention measurements with a total sample of 13,216 participants to identify the effectiveness of the interventions and to examine the impact of underlying, predefined factors.

In line with prior research the results of the meta-analysis partially support the medium-to-low effectiveness of digital mental health promoting programs. In particular, small effects regarding a decrease of anxiety and an increase of well-being were identified, a finding which is consistent with previous research (Clarke et al., 2015; Harrer et al., 2019; Sevilla-Llewellyn-Jones et al., 2018). This seems particularly relevant given that anxiety is one of the most prevalent mental disorders in childhood and adolescence (Polanczyk et al., 2015). While Noh and Kim (2022) did not find beneficial results with respect to anxiety prevention, they suggest that this is due to the fact that their studies examined studies of general and at-risk populations whereas, for example, Sevilla-Llewellyn-Jones et al. (2018) examined studies of clinical populations where there is likely to be greater room for improvement in the related mental health domain (Noh & Kim, 2022).

After outlier-removal, small effects were also detected relating to the promotion of protective individual factors, including self-esteem, self-compassion, or help-seeking behavior. Contrary to the results of previous research (Clarke et al., 2015; Harrer et al., 2019; Sevilla-Llewellyn-Jones et al., 2018), no significant effects were found for depressive symptoms, stress, externalizing symptoms (e.g., hyperactivity, behavioral problems), and internalizing symptoms (e.g. loneliness, rumination, emotional difficulties).

When examining the impact of underlying predefined factors, the analysis showed that school-based interventions with consistent adherence, low levels of attrition and some level of professional support and guidance, were found to be most effective. These findings confirm previous research showing that schools are an appropriate setting for promoting and supporting mental health in children and adolescents (e.g., Cilar et al., 2020). A school-based setting, some level of professional support, and low levels of attrition were found to be the most beneficial concerning anxiety relief. A school-based setting was also found to be most effective in the improvement of internalizing disorders, besides a mixed setting, which generated modest beneficial effects too. Consistent adherence was shown to have the greatest effects on depressive symptoms and the administration of some level of guidance, in contrast to considerable or no guidance, was seen to provide the most benefit in the enhancement of protective factors. This result is partially consistent with the findings of Sevilla-Llewellyn-Jones et al. (2018) who identified studies in which engagement increased and outcomes improved when adherence was augmented by, for example, a guided diagnostic procedure, or the provision of feedback by a mental health professional (Kauer et al., 2012).

Even though individual attrition rates were up to 58% in the current sample, the mean attrition rate (*M* = 25.2%), relating to the most recent time point, remained below that found in the literature (*M*_weighted_ = 31) for internet-based treatment programs (Melville et al., 2010). However, the level of drop out in the current sample was noticeable (>20%) for about half the sample. This should not be neglected as high attrition rates may lead to underestimating the impact of the intervention (Eysenbach, 2005). High attrition is nothing new to school-based research and significant attention and effort has to be channeled into obtaining teachers’ and students’ compliance in order to maintain as many participants as possible (Calear et al., 2016). As mentioned above, one facilitator of efficacy is the level of guidance, where *some*, rather than *none* or *substantial guidance* was found to be most beneficial. This finding might be related to participants’ choice and higher motivation and engagement (Burckhardt et al., 2015), which is assumed to be present whenever there is a balance between sufficient instruction and freedom to choose ways of engagement. The presence of considerable guidance in a program might even be a barrier to effective intervention implementation, especially when guidance, and thus time and effort, is required by teachers (Fridrici & Lohaus, 2009). Guidance features thus need to be designed in such a way that no additional strain is placed on teachers during program implementation. At the same time, the age of the target group must be carefully considered, and more emphasis must be placed on age level when designing an intervention, as age has been found to significantly moderate its effects (e.g., Osborn et al., 2020; Sousa et al., 2020). It is advisable to focus on a narrow age range rather than a broad one, as the needs of older and younger adolescents may differ considerably in terms of the pace or challenge of an activity (Egan et al., 2021).

Another potential barrier with respect to detecting efficacy relates to the adequate choice of control condition. In three out of seven studies that used an alternative intervention program in the current sample, both conditions led to improvements in outcome measures (and non-significant between-group effects) and possibly made intervention effects harder to detect. Considerable discernment is therefore called for, particularly when opting for an alternative intervention method in place of the more common (waitlist) control group.

Additionally, the participants’ baseline level can also be seen as a potential barrier when detecting efficacy. Participants reporting higher levels of distress showed a higher increase in targeted outcome measures (Kauer et al., 2012; Osborn et al., 2020; Puolakanaho et al., 2019). Similarly, it has been argued that low levels of distress may be a reason for ineffective interventions (Bohleber et al., 2016; Calear et al., 2016; Kenny et al., 2020; van Vliet & Andrews, 2009). Thus, higher baseline levels of distress often led to a greater benefit of the intervention. Or, in other words, low baseline levels of distress may help explain why efficacy was not found in some studies. This was in line with results of previous studies that found greater benefits for targeted, compared to universal interventions (Feiss et al., 2019; Werner-Seidler et al., 2017), using more specific inclusion criteria. Feiss et al. (2019) found greater stress reduction and Werner-Seidler et al. (2017) greater reduction of depressive symptoms for targeted interventions compared to universal interventions. The establishment of appropriate inclusion criteria can therefore help target that part of the population which may benefit most from an intervention. Whatever the case, recent research shows that even individuals with mild, subclinical symptom levels may benefit from help (Ruscio 2019). Hence, universal interventions should not be disregarded, especially since adolescence is a vulnerable time when mental health problems often present themselves for the first time (Solmi et al., 2022). Therefore, in addition to targeted interventions, it is important to provide universal programs, preferably those that are adapted to the individual needs of young people in order to prevent initial disorder development.

Given that classrooms, as well as society overall, are increasingly characterized by diversity awareness, it was surprising that only two studies focused on minorities, namely sexual gender minority youth (Egan et al., 2021) and youth who identified as American Indian and Alaskan native (Craig Rushing et al., 2021), and only three studies reported on non-binary gender identification options. In their study, Craig Rushing et al. (2021) used a positive representation of native youth in both intervention conditions (alternate and intervention), and both groups showed improvement in mental health outcomes, such as resilience, coping or self-esteem. This demonstrates the importance of culturally relevant content and minority representation in the interventions themselves. This is a clear indication that future interventions should be designed with greater diversity awareness in mind.

Several limitations were detected in the process of reviewing and analyzing the data which have to be considered when interpreting the reported findings. One important limitation, especially regarding the subgroup analysis, is the high level of heterogeneity among the studies, which probably relates back to the creation of outcome clusters that were not directly derived from the original authors’ intentions. However, such clustering was necessary for the meta-analytic analysis and has also been found to be a sound approach in prior research (Harrer et al., 2019). Another limitation was the failure to compare universal and targeted interventions, as has been done in other systematic reviews and meta-analyses (Feiss et al., 2019; Werner-Seidler et al., 2017). However, the relatively small number (5) and high variability in mental health domains of the targeted interventions included in the present study did not allow for adequate statistical analysis. The plethora of mental health domains that are targeted within the mental health promoting interventions can also be seen as a limitation, as this could result in some discrepancy in the measurements of the outcomes used in the meta-analysis. This may be traced back to the nature of the concept itself which entailed the application of a broad range of outcome measures in many of the studies reviewed. A more targeted focus on specific mental health domains is therefore advisable in future research. Another associated limitation, and mentioned earlier, is the high heterogeneity observed in the intervention effects. In addition to the clustering issue, the heterogeneity could also be due to the wide variation in content, setting and length of the interventions. Two further limitations common in mental health research were also observed in the current sample, i.e., a sole reliance on self-reported measures, and the non-blinding of participants. These were mainly responsible for the relatively unfavorable results regarding the risk of bias, where one quarter of the RCTs raised some concerns, and a little less than half of the CTs raised serious concerns.

Based on the results of the review and meta-analysis, there are several important points that need to be taken into consideration for future studies. First, moderating variables must be considered in efficacy analyses, as they can have a significant impact on the success of an intervention. Setting, level of guidance and level of professional support were found to be particularly influential in the current study. The provision of adequate support and guidance for participants could also greatly improve the outcomes of prevention studies. It is recommended that support and guidance be offered in an adaptive manner, i.e., one which is tailored to the needs of the participants. Thus, special attention should be given to such moderating variables in the design of future studies. Second, two other important factors that influence the effectiveness of the intervention are adherence to the intervention protocol and the attrition rate. It is critical to provide adequate opportunities for engagement to keep participation high and dropout rates low. This entails considerable effort in study implementation. Therefore, more attention should be paid to implementation quality in the future, and interventions with smaller groups but higher implementation quality should be preferred over studies with large numbers of participants. Third, interventions employing diversity-sensitive design and content are critical in meeting the needs of all youth. Fourth, specific mental health domains should be addressed as part of the interventions in order to reduce the broad range of outcomes and to obtain meaningful results concerning impact. Finally, as only 13 of the interventions studied here were available to students, and even fewer (9) were free and accessible for all, it is crucial that further research look just as closely at maintaining and promoting the availability of an intervention as at intervention development.

## Conclusion

Given the alarming number of young people suffering from mental health issues, there is a great need for easily accessible mental health promoting tools. These tools are increasingly being made available digitally, and in line with rapid technological development, the field of digital mental health tools is growing fast. Thus, an up-to-date look at these programs and their efficacy is clearly needed. This systematic review and meta-analysis provides such an overview and offers insight into the effectiveness, barriers and facilitators relating to digital evidence-based mental health programs for youth aged 11 to 18 years. Even though results have to be interpreted with caution, the findings support previous research in that digital interventions have the potential to promote adolescent mental health. Small effects were found for well-being, anxiety and protective factors (e.g. help-seeking behavior). Important factors in tool efficacy include the setting, levels of guidance and support and intervention adherence. It was found that a school-based setting, some level of guidance and professional support, and consistent adherence to the intervention were most beneficial. Future research should pay particular attention to these moderating factors, to a diversity-sensitive design and content, as well as to a sustained availability of the tools developed.

## Supplementary information

Supplementary Material 1

Supplementary Material 2

Supplementary Material 3

Supplementary Material 4

Supplementary Material 5
