# Supplementary material for: Interventions with Digital Tools for Mental Health Promotion among 11–18 Year Olds: A Systematic Review and Meta-Analysis
Source: J Youth Adolesc. 2023 Feb 8;52(4):754–79. doi: 10.1007/s10964-023-01735-4 (PMC9907880; doi:10.1007/s10964-023-01735-4)
Supplement: Supplementary file 1 — Supplementary Material 1 [file 10964_2023_1735_MOESM1_ESM.docx]

**Supplementary Material 1**

*Clusters*

| **Cluster** | **Study** | **Scale** |
| --- | --- | --- |
| Well-being (N = 8) |  |  |
|  | Calear et al., 2016 | WEMBS: Well-Being |
|  | De la Barrera et al., 2021 | SPANE: Affect Balance |
|  | Edridge et al., 2020 | SWEMBS: Well-Being |
|  | Edridge et al., 2020 | SWEMBS: Well-Being |
|  | Haug et al., 2021 | WHO-5: Well-Being |
|  | Manicavasagar et al., 2014 | SWEMBS: Well-Being |
|  | O’Dea et al., 2020 | SWEMBS: Well-Being |
|  | Osborn et al, 2020 | SWEMBS: Well-Being |
| Anxiety (N = 11) |  |  |
|  | Calear et al., 2016 | SCAS: Generalized Anxiety |
|  | **Calear et al., 2016** | **GAD-7: Generalized Anxiety** |
|  | Calear et al., 2016 | SAS-A: Social Anxiety |
|  | Calear et al., 2016 | CASI: Anxiety Sensitivity |
|  | Egan et al., 2021 | GAD-C: Anxiety |
|  | Kauer et al., 2012 | DASS-21: Anxiety |
|  | Malboeuf-Hurtubise et al., 2021 | BASC-II: Anxiety |
|  | Manicavasagar et al. (2014) | DASS-21: Anxiety |
|  | **O’Dea et al. (2020)** | **SCASAGAD: Generalized Anxiety** |
|  | O’Dea et al. (2020) | SCASSAD: Separation Anxiety |
|  | O’Dea et al. (2021) | GAD-7: Anxiety |
|  | Osborn et al. (2020) | GAD-7: Anxiety |
|  | Perkins et al. (2021) | RCADS-25: Anxiety |
|  | Schleider et al. (2020) | SPI: Social anxiety |
|  | Zheng et al. (2021) | SCAS: Anxiety |
| Depressive Symptoms (N = 11) |  |  |
|  | Calear et al., 2016 | CES-D: Depressive Symptoms |
|  | Egan et al., 2021 | PHQ-9: Depressive Symptoms |
|  | Kauer et al., 2012 | DASS-21: Depression |
|  | Malboeuf-Hurtubise et al., 2021 | BASC-III: Depression |
|  | Manicavasagar et al. (2014) | DASS-21: Depression |
|  | O’Dea et al. (2020) | PHQ-9: Depressive Symptoms |
|  | O’Dea et al. (2021) | CES-DC: Depressive Symptoms |
|  | Osborn et al. (2020) | PHQ-8: Depressive Symptoms |
|  | Perkins et al. (2021) | RCADS-25: Depression |
|  | Santor et al. (2007) | BDI: Depression |
|  | Schleider et al. (2020) | SMFQ: Depressive Symptoms |
| Stress (N = 7) |  |  |
|  | Bohleber et al., 2016 | TICS: Chronic Stress |
|  | **Fridrici & Lohaus, 2009** | **SSKJ 3-8: Psychological stress-symptoms** |
|  | Fridrici & Lohaus, 2009 | SSKJ 3-8: Physical stress symptoms |
|  | Fridrici & Lohaus, 2009 | SSKJ 3-8: Stress vulnerability |
|  | Haug et al., 2021 | SJS: Perceived Stress |
|  | Kauer et al., 2012 | DASS-21: Stress |
|  | Manicavasagar et al. (2014) | DASS-21: Stress |
|  | Puolakanaho et al., 2019 | Single question: Overall stress |
|  | **Puolakanaho et al., 2019** | **HBSC: School stress** |
|  | Sousa et al. (2020) | ALP: stress management |
| Protective Factors (N = 11) |  |  |
|  | **De la Barrera et al. (2021)** | **RSES: Self-Esteem** |
|  | De la Barrera et al. (2021) | TMMS-24: Attention (Emotional Intelligence) |
|  | De la Barrera et al. (2021) | TMMS-24: Clarity (Emotional Intelligence) |
|  | De la Barrera et al. (2021) | TMMS-24: Repair (Emotional Intelligence) |
|  | Edridge et al., 2020 | Youth Empowerment Scale: Self-efficacy |
|  | Edridge et al., 2020 | Youth Empowerment Scale: Self-efficacy |
|  | Egan et al. (2021) | MD Scale of Perceived SE: Social self-efficacy |
|  | **Egan et al. (2021)** | **General Help-Seeking-Questionnaire: Help-seeking intentions for personal or emotional problems** |
|  | Egan et al. (2021) | MD Scale of Perceived SE: Parental and community support |
|  | Haug et al. (2021) | ICQ-10: Social skills |
|  | **O'Dea et al. (2020)** | **GHSQ: Help-Seeking** |
|  | O'Dea et al. (2020) | SEQ-C: Self-Efficacy |
|  | O'Dea et al. (2021) | GHSQ: General Help-Seeking |
|  | **Osborn et al. (2020)** | **EPOCH: Optimism** |
|  | Osborn et al. (2020) | EPOCH: Happiness |
|  | Perkins et al. (2021) | IPTQ: Personality Mindset |
|  | Perkins et al. (2021) | SCS-SF: Self-compassion |
|  | **Perkins et al. (2021)** | **RSES: Self-esteem** |
|  | Santor et al. (2007) | Wanted help for problems |
|  | Sousa et al. (2020) | ALP: Positive life perspective |
| Internalizing Symptoms (N = 10) |  |  |
|  | **De la Barrera et al., 2021** | **SDQ: Emotional symptoms** |
|  | De la Barrera et al., 2021 | SDQ: Peer Problems |
|  | **Douma et al., 2021** | **CBCL: Internalizing Behavioral Problems** |
|  | Douma et al., 2021 | YSR: Internalizing Behavioral Problems |
|  | Douma et al., 2021 | PedsQL: Emotional Functioning |
|  | Edridge et al., 2020 | M&MS: Emotional Difficulties |
|  | Edridge et al., 2020 | M&MS: Emotional Difficulties |
|  | Egan et al., 2021 | ULS: Loneliness |
|  | Kauer et al., 2012 | RRS: Rumination |
|  | **O'Dea et al. (2020)** | **DQ5: Psychological Distress** |
|  | O'Dea et al. (2020) | TBS: Belongingness (Loneliness) |
|  | O'Dea et al. (2021) | DQ5: Psychological Distress |
|  | Santor et al. (2007) | Mood Problem Subscale: Mood Problems |
|  | Zheng et al. (2021) | PROMIS: Sleeping problems |
| Externalizing symptoms (N = 6) |  |  |
|  | **De la Barrera et al., 2021** | **SDQ: Behavioral problems** |
|  | De la Barrera et al., 2021 | SDQ: Hyperactivity |
|  | **Douma et al., 2021** | **CBCL: Externalizing Behavioral Problems** |
|  | Douma et al., 2021 | YSR: Externalizing Behavioral Problems |
|  | Edridge et al., 2020 | M&MS: Behavioral Difficulties |
|  | Edridge et al., 2020 | M&MS: Behavioral Difficulties |
|  | Malboeuf-Hurtubise et al., 2021 | BASC-II: Inattention |
|  | **Malboeuf-Hurtubise et al., 2021** | **BASC-II: Hyperactivity** |
|  | Schleider et al. (2020) | Measure of conduct problem behavior: Conduct problems/antisocial behavior |
