# Supplementary material for: Interventions with Digital Tools for Mental Health Promotion among 11–18 Year Olds: A Systematic Review and Meta-Analysis
Source: J Youth Adolesc. 2023 Feb 8;52(4):754–79. doi: 10.1007/s10964-023-01735-4 (PMC9907880; doi:10.1007/s10964-023-01735-4)
Supplement: Supplementary file 2 — Supplementary Material 2 [file 10964_2023_1735_MOESM2_ESM.docx]

**Supplementary Material 2**

*Availability of interventions*

| # | Study | Intervention availability |
| --- | --- | --- |
| 1 | Bohleber et al., 2016 | Not found |
| 2 | Burckhardt et al., 2015 | Yes, free: <https://www.biteback.org.au/> |
| 3 | Calear et al., 2016 | Yes, with fee: <https://ecouch.com.au/info/new_ecouch> |
| 4 | Craig Rushing et al., 2021 | Yes, free: <https://www.healthynativeyouth.org/curricula/brave/> |
| 5 | De la Barrera et al., 2021 | Yes, free: <https://play.google.com/store/apps/details?id=com.uvUpv.EmoTIC_Demo&hl=en&gl=US> |
| 6 | Douma et al., 2021 | No (live session) |
| 7 | Edridge et al., 2020 | Yes, free: <https://appsonwindows.com/apk/7822784/> |
| 8 | Egan et al., 2021 | Not found: Download link was sent to participants - no website or online presence found |
| 9 | Fridrici & Lohaus, 2009 | Not found: error for provided link ([www.snake-training.de](http://www.snake-training.de)) |
| 10 | Haug et al., 2021 | Yes, free: <https://www.smartcoach.info/> |
| 11 | Huppert & Johnson, 2010 | No (live sessions) |
| 12 | Kauer et al., 2012 | Not found (for use with GPs) |
| 13 | Kenny et al., 2020 | Not found |
| 14 | Kutok et al., 2021 | Not found |
| 15 | Malboeuf-Hurtubise et al., 2021 | No (live sessions) |
| 16 | Manicavasagar et al., 2014 | Yes, free: <https://www.biteback.org.au/> |
| 17 | O’Dea et al., 2020 | Yes: <https://play.google.com/store/apps/details?id=au.org.blackdoghealth.weclick&hl=en&gl=US>  Available for participants of study and for students in Australia |
| 18 | O’Dea et al., 2021 | Yes: service in schools in Australia |
| 19 | Osborn et al., 2020 | Yes, free: <https://thrive-online.shamiri.institute/> |
| 20 | Perkins et al., 2021 | Not found |
| 21 | Puolakanaho et al., 2019 | Not found |
| 22 | Santor et al., 2007 | Not found: error for provided link ([www.yoomagazine.net](http://www.yoomagazine.net)) |
| 23 | Schleider et al., 2020 | Yes, free: <https://www.projectgrowingminds.com/intro> |
| 24 | Sousa et al., 2020 | Yes, free: <https://apkcombo.com/teenpower/pt.ipleiria.teenpowerapp/> |
| 25 | Van Vliet & Andrews, 2009 | Yes, free at first, then to pay per student: <https://ourfutures.education/modules-we-offer> |
| 26 | Yuan, 2021 | Not found (recordings) |
| 27 | Zheng et al., 2021 | Not found |
