# Supplementary figures and images for: Interventions with Digital Tools for Mental Health Promotion among 11–18 Year Olds: A Systematic Review and Meta-Analysis
Source: J Youth Adolesc. 2023 Feb 8;52(4):754–79. doi: 10.1007/s10964-023-01735-4 (PMC9907880; doi:10.1007/s10964-023-01735-4)

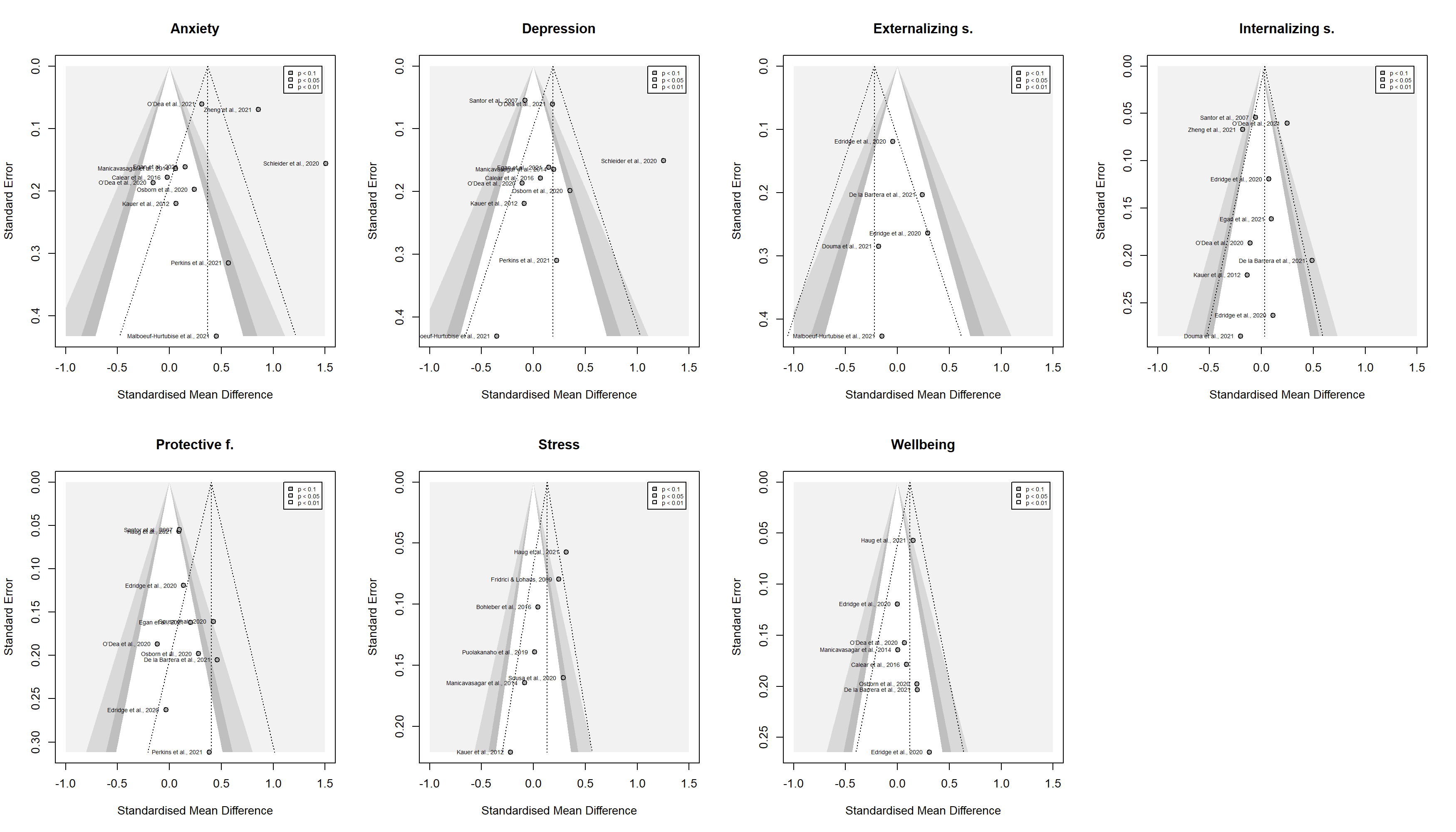

Supplement: Supplementary file 3 — Supplementary Material 3 [file 10964_2023_1735_MOESM3_ESM.png]

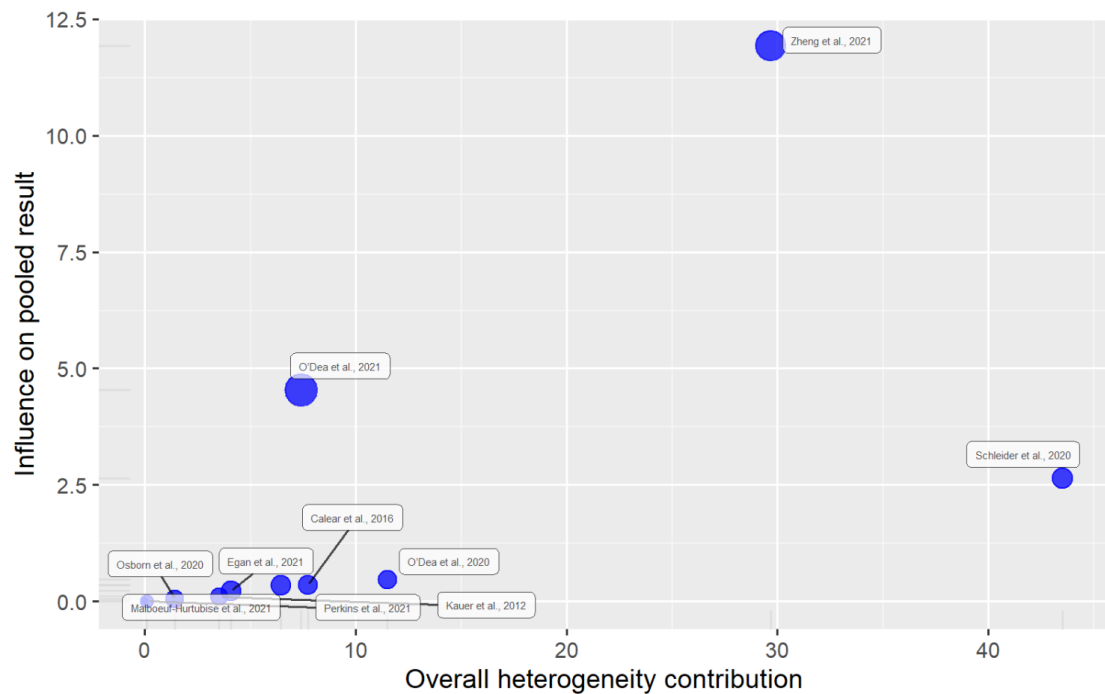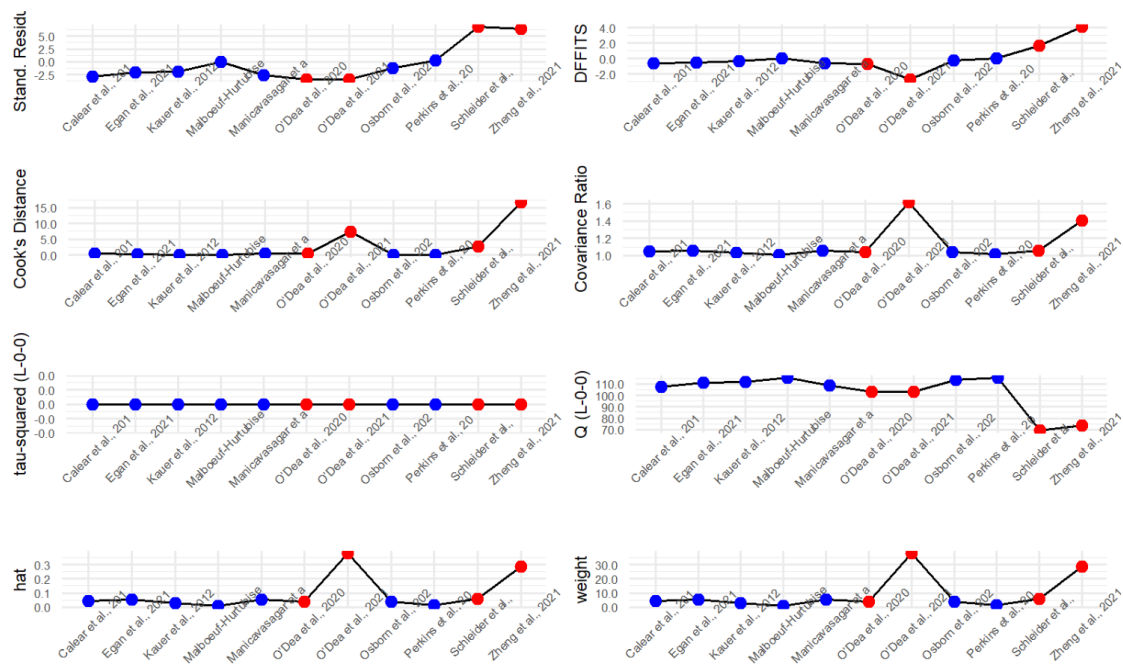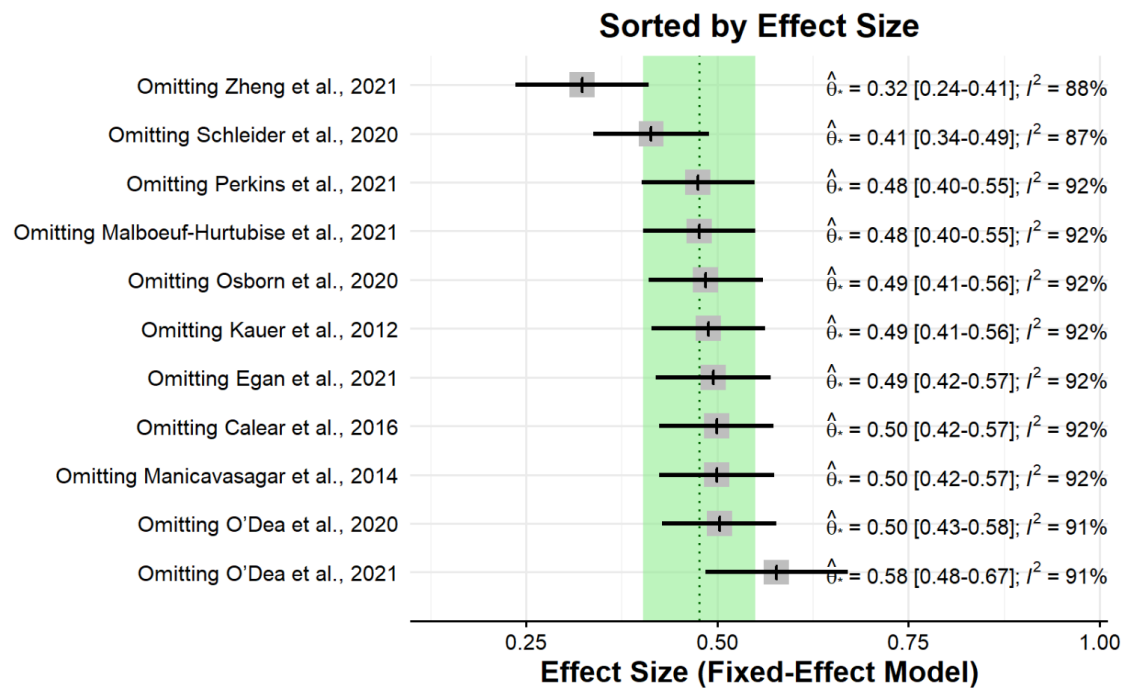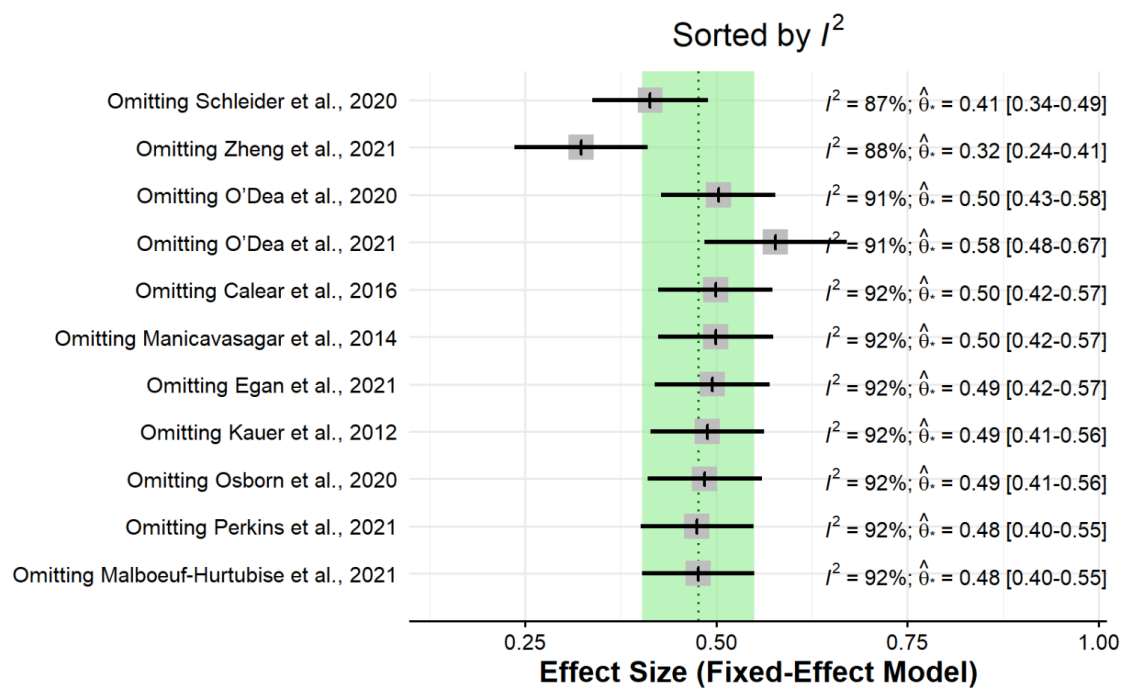

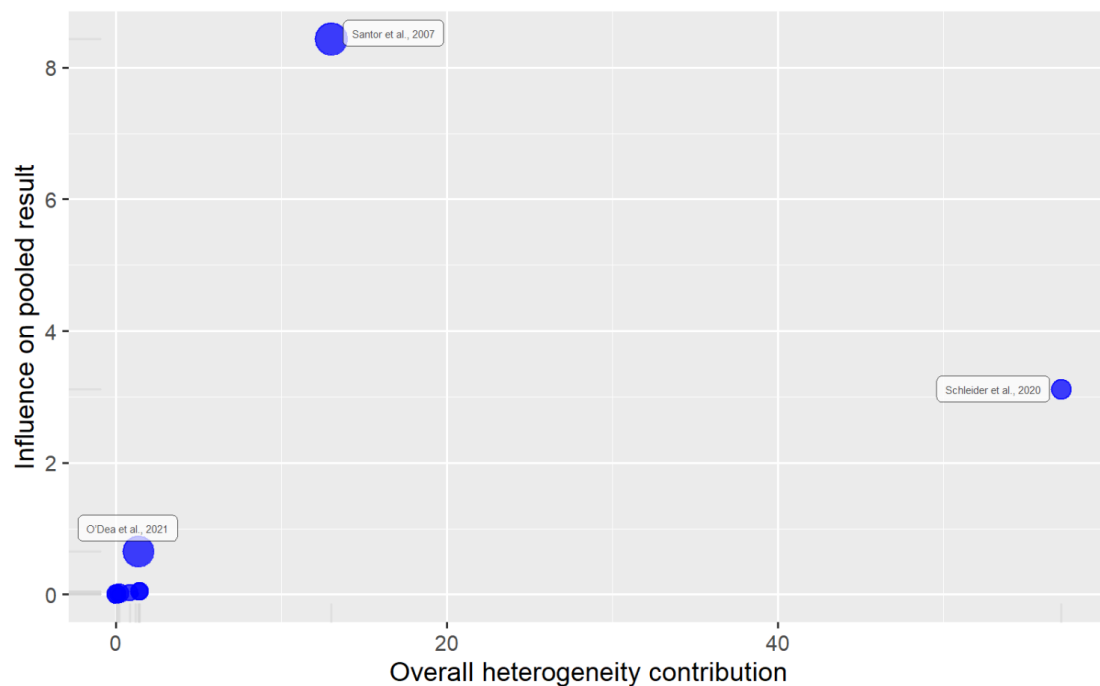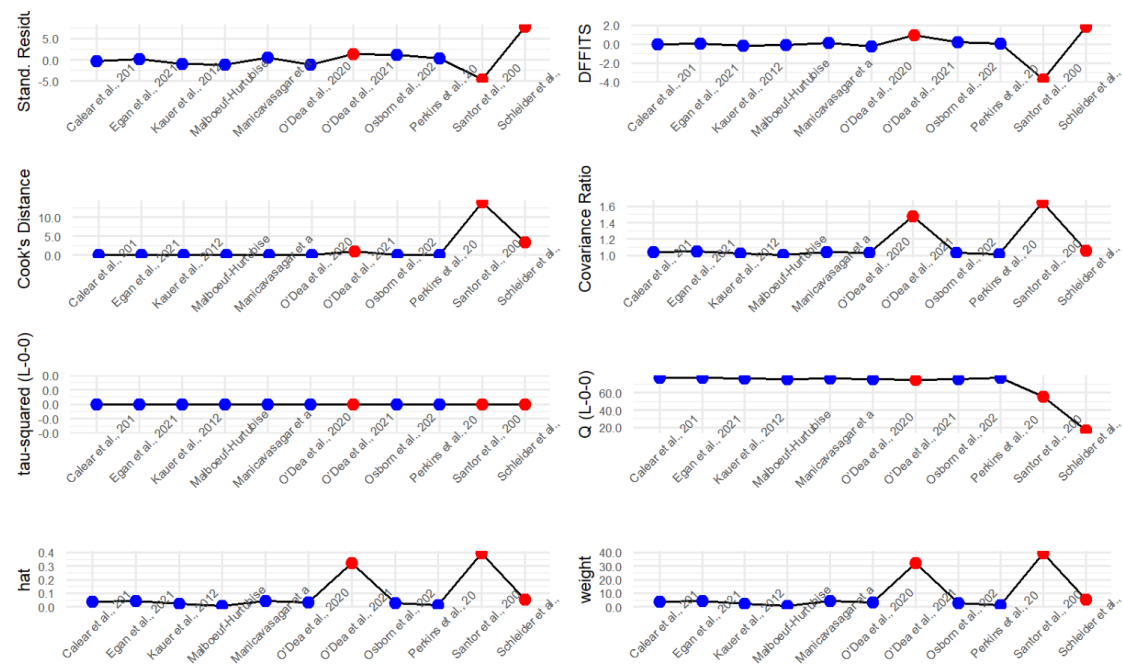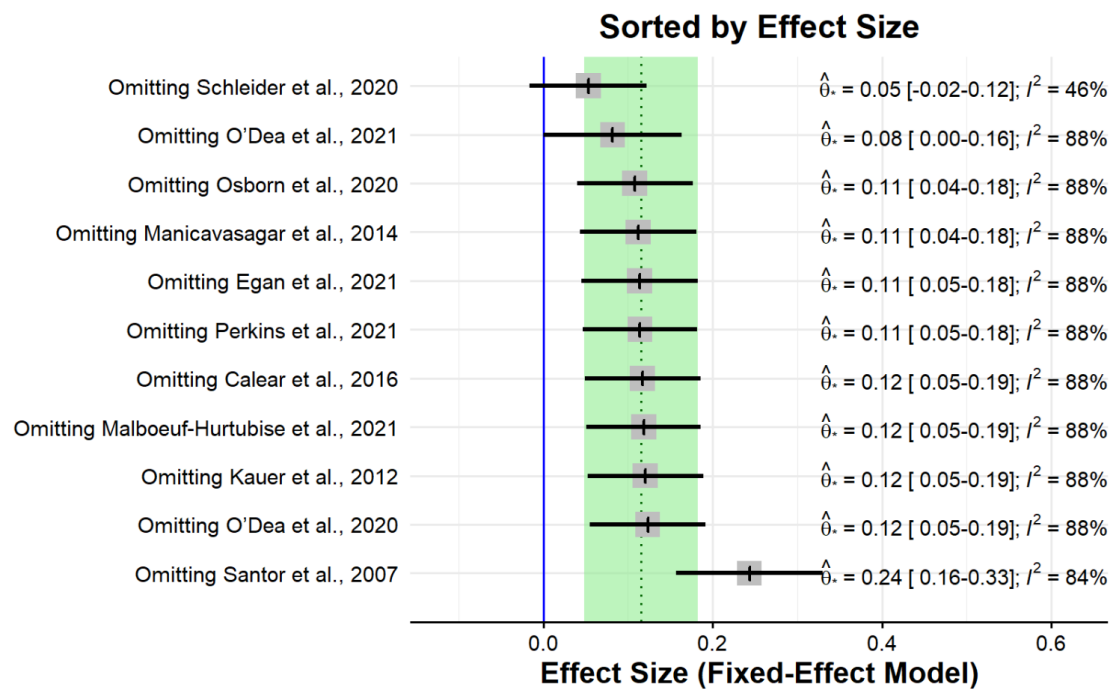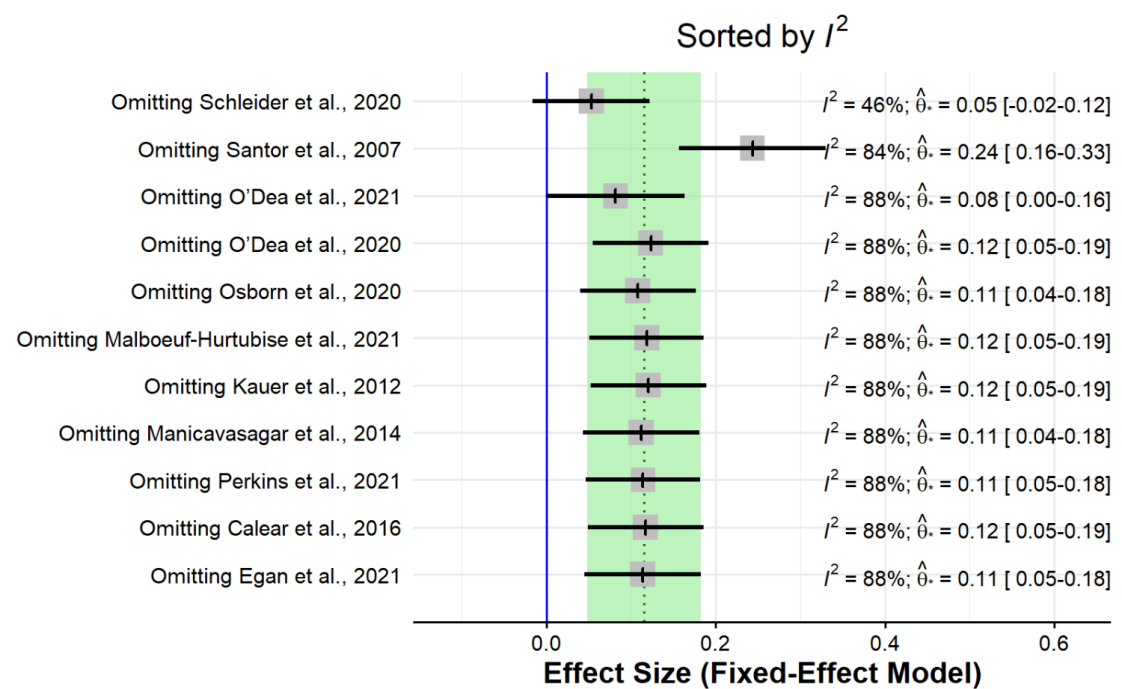

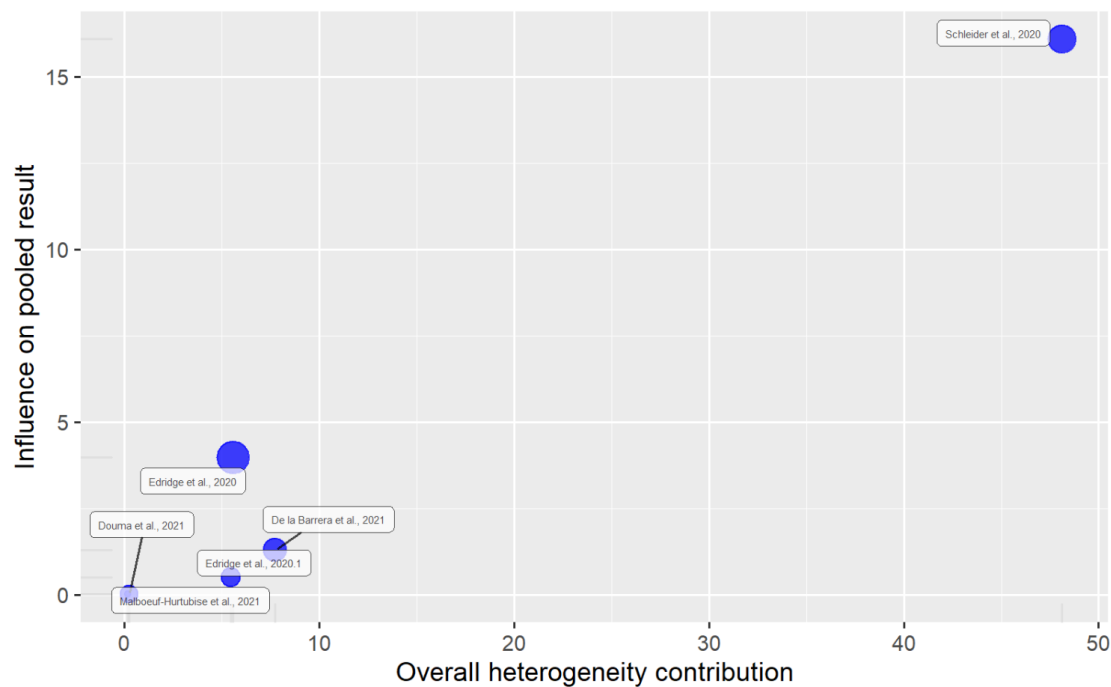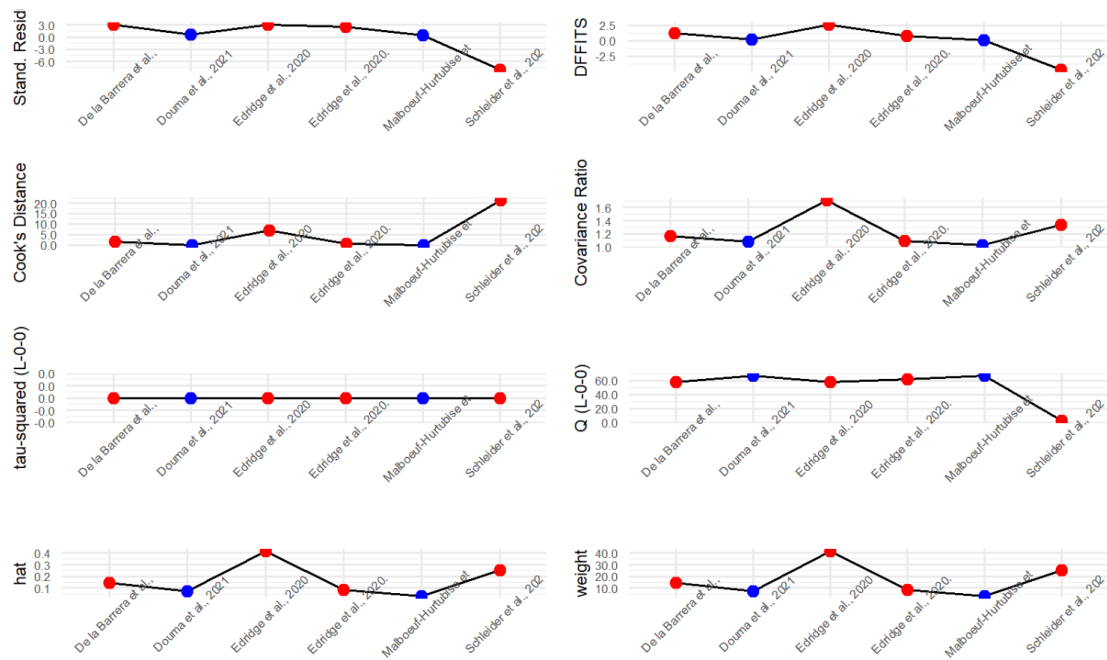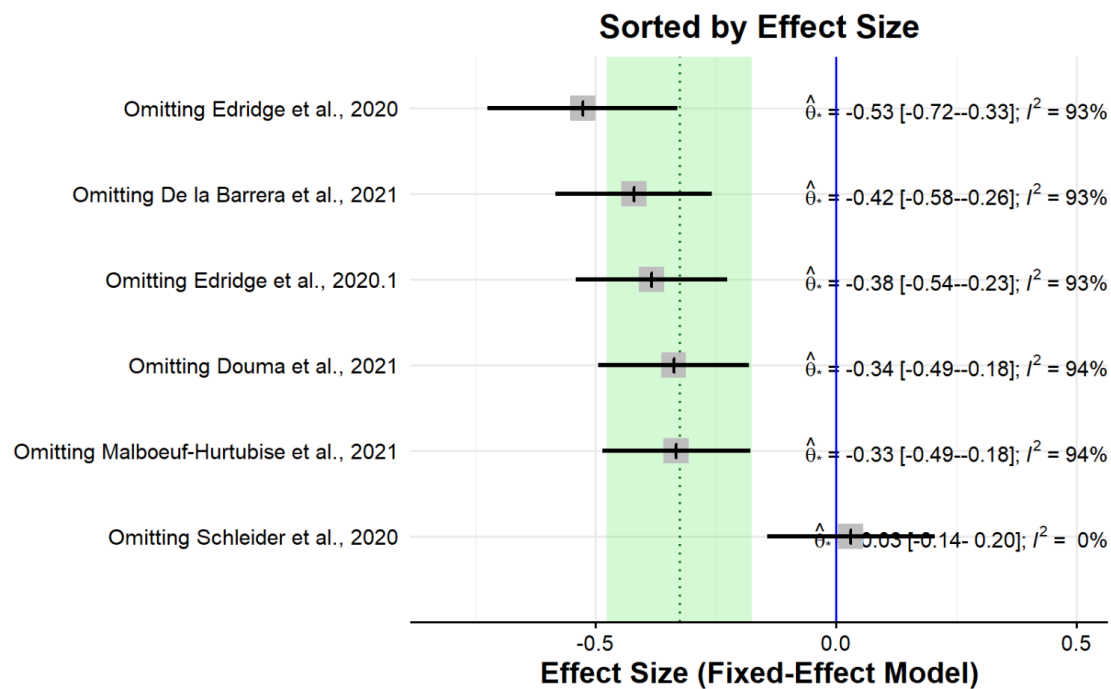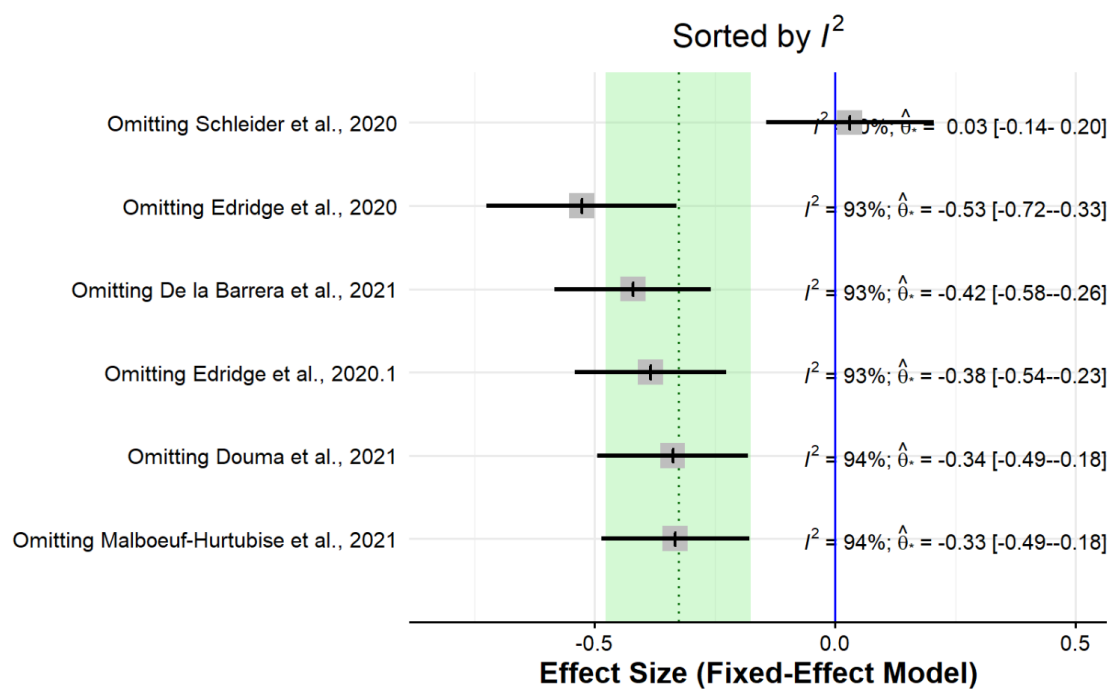

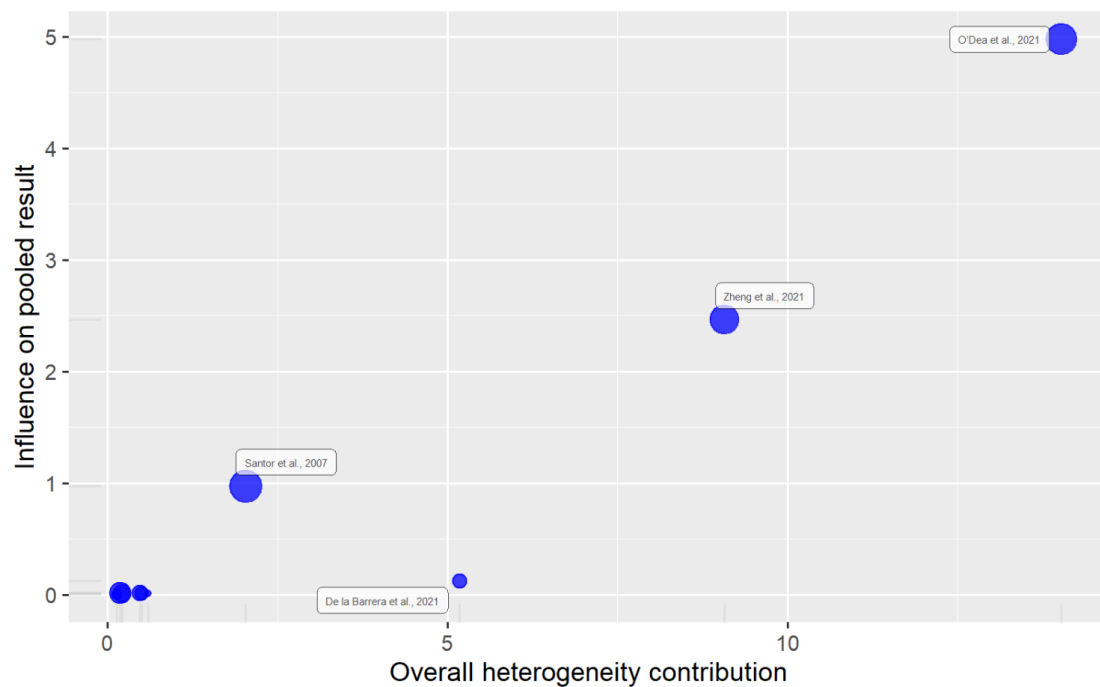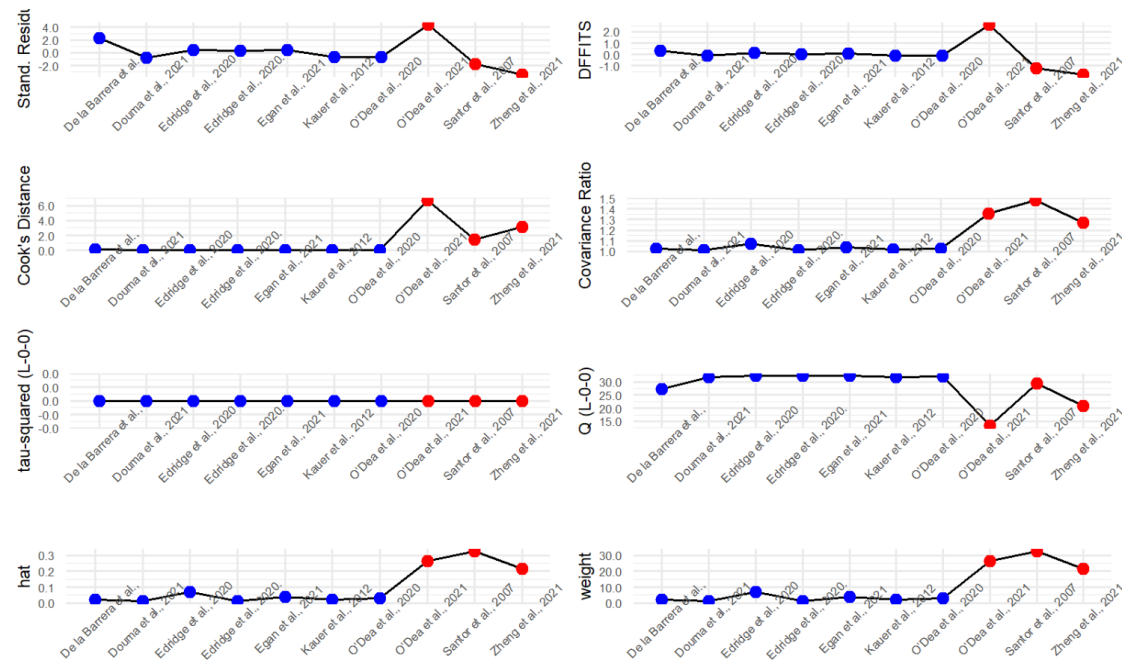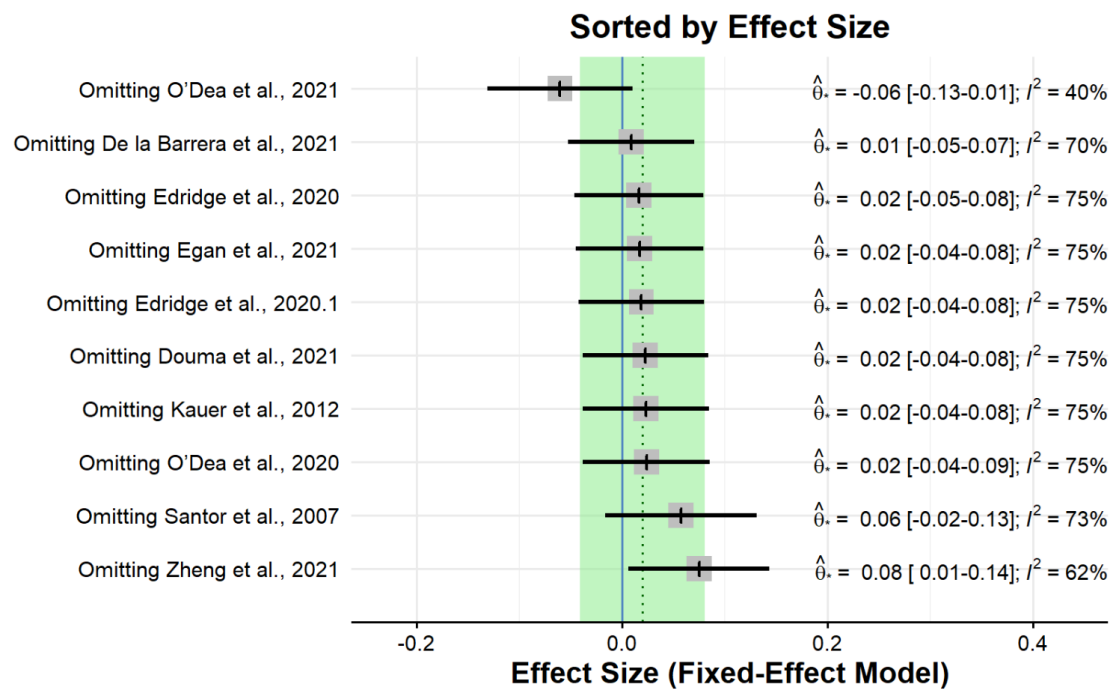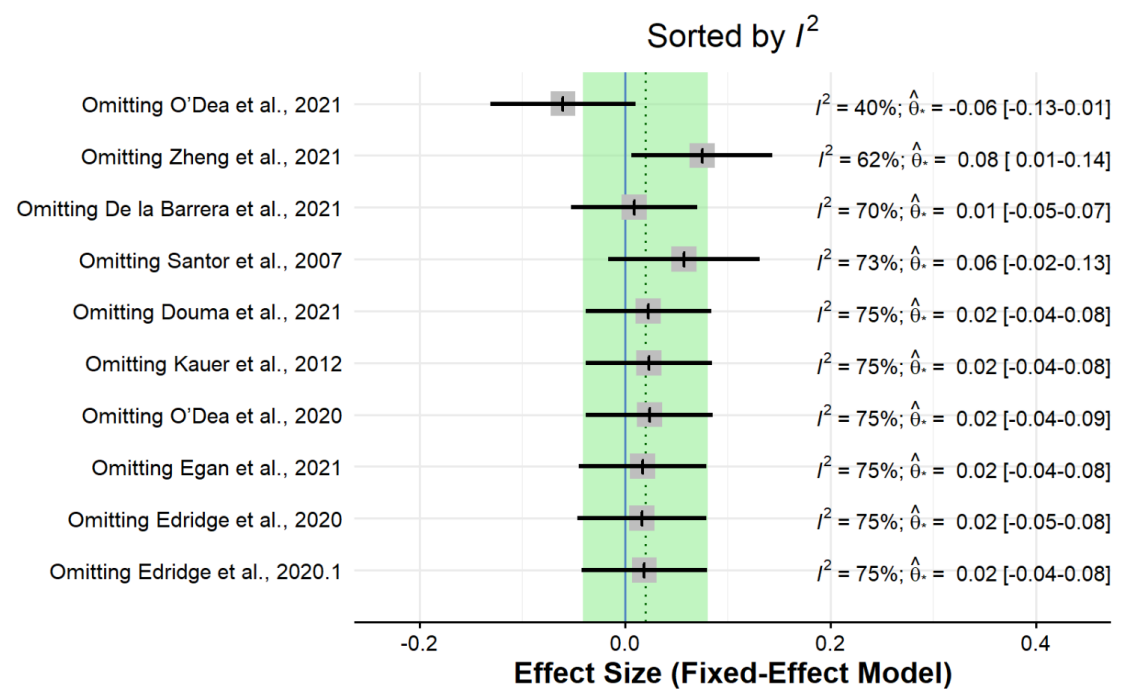

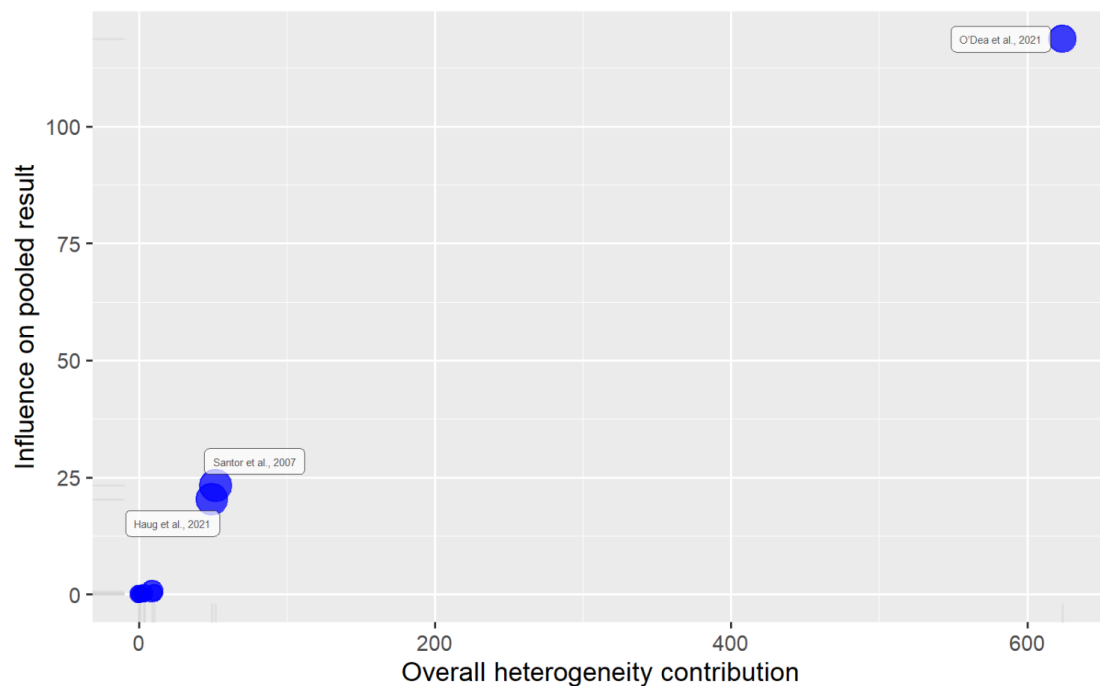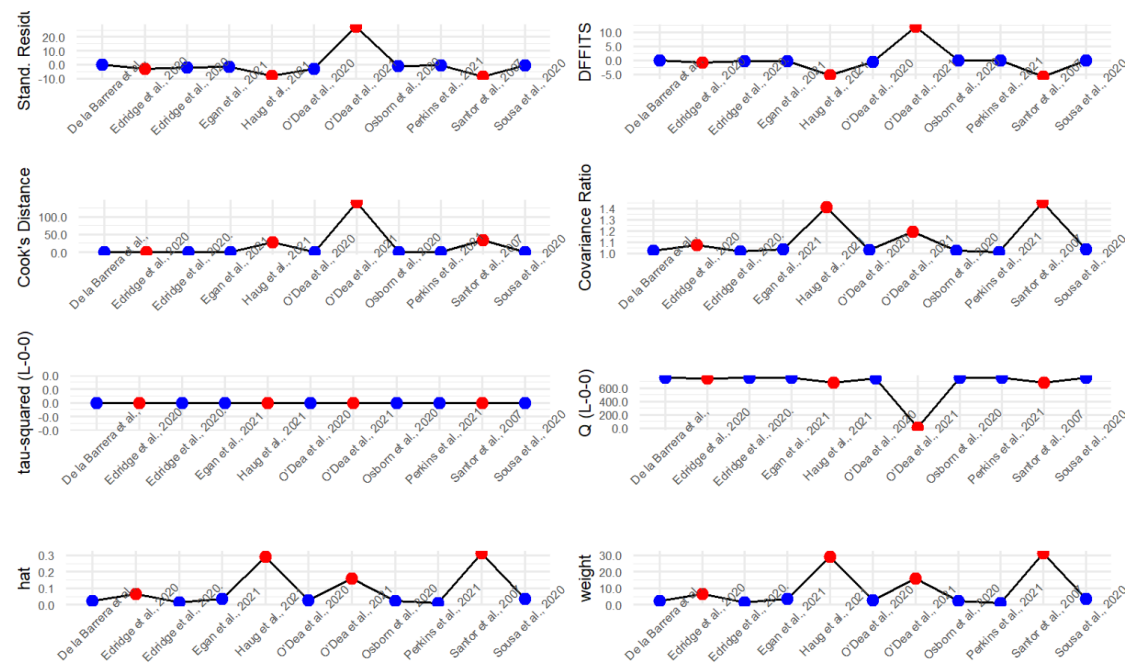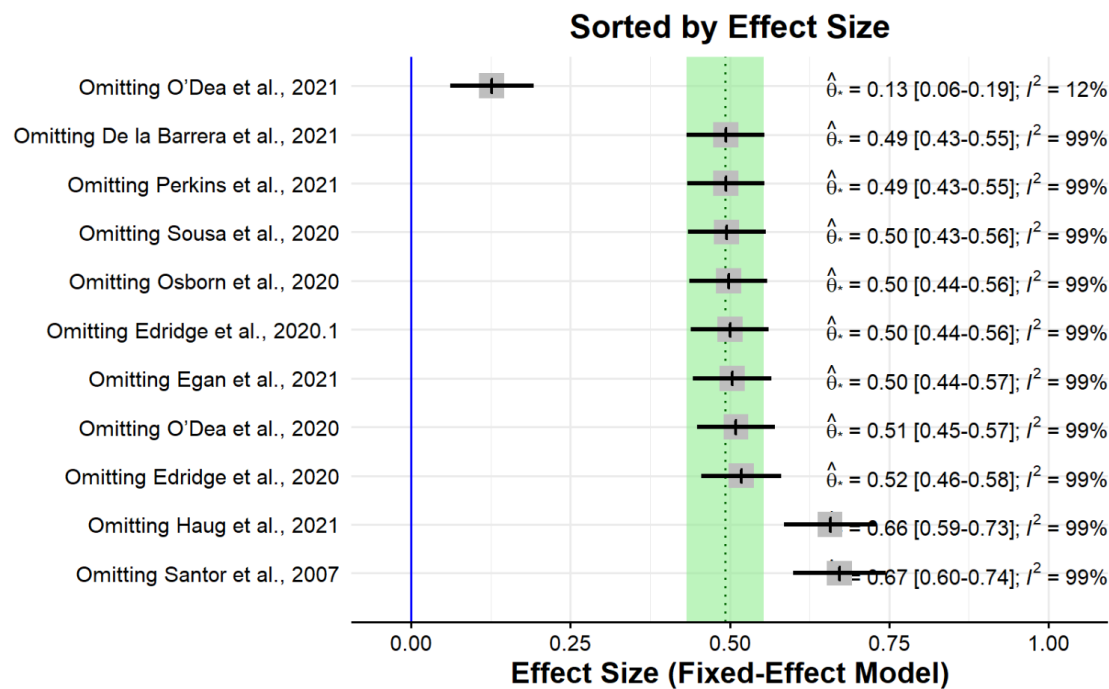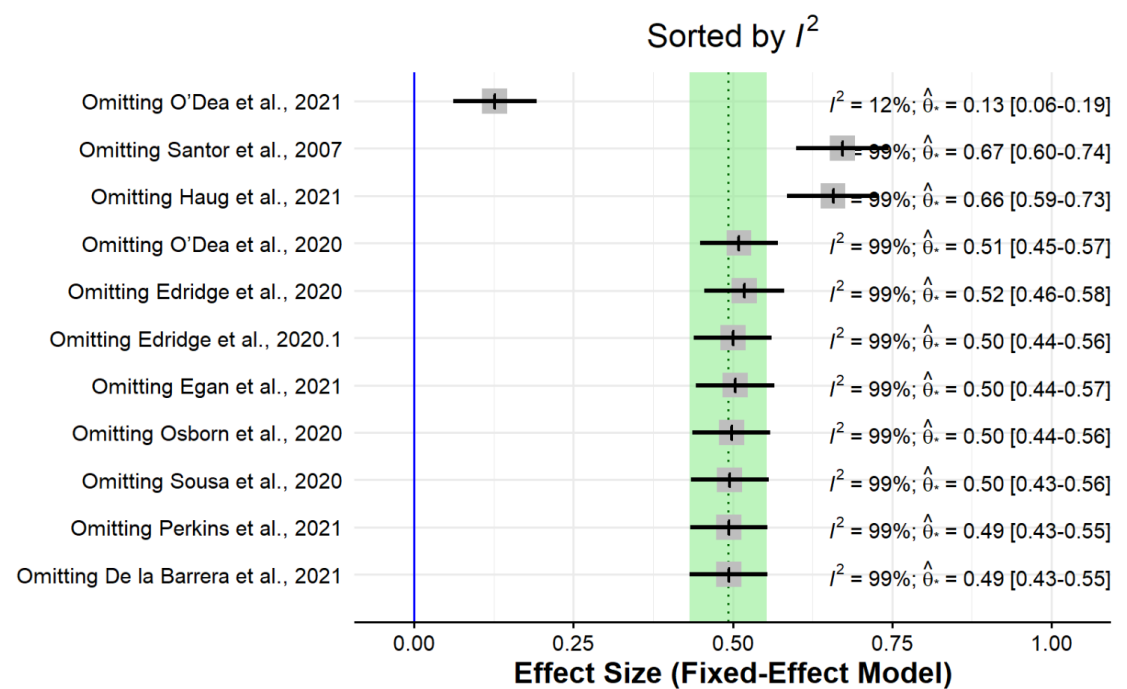

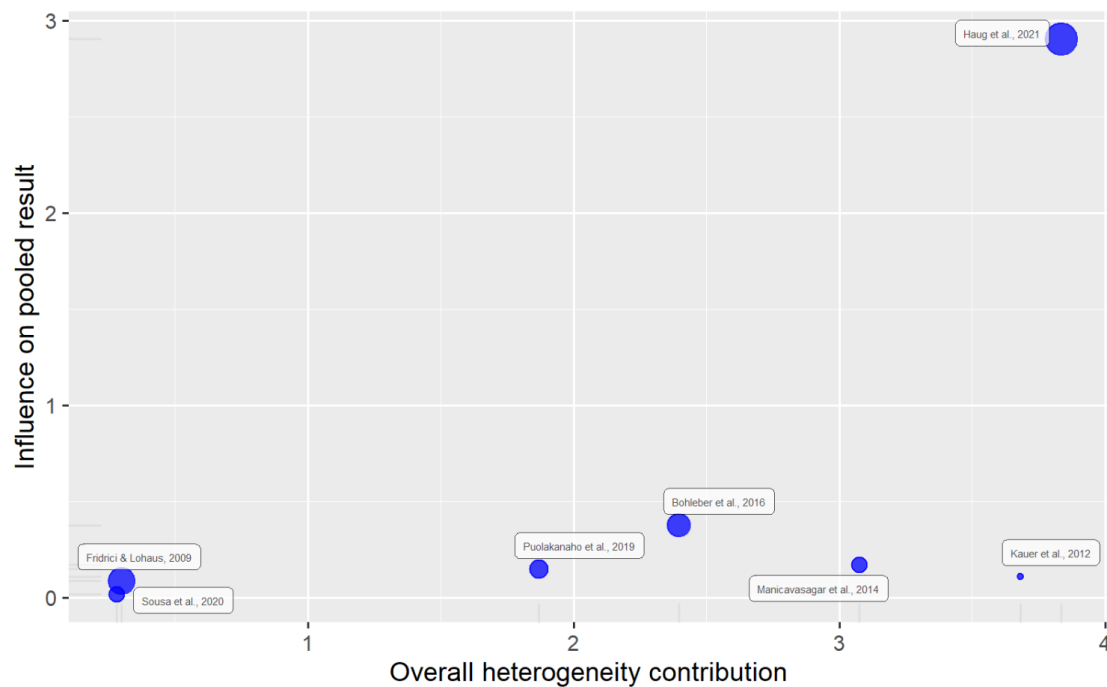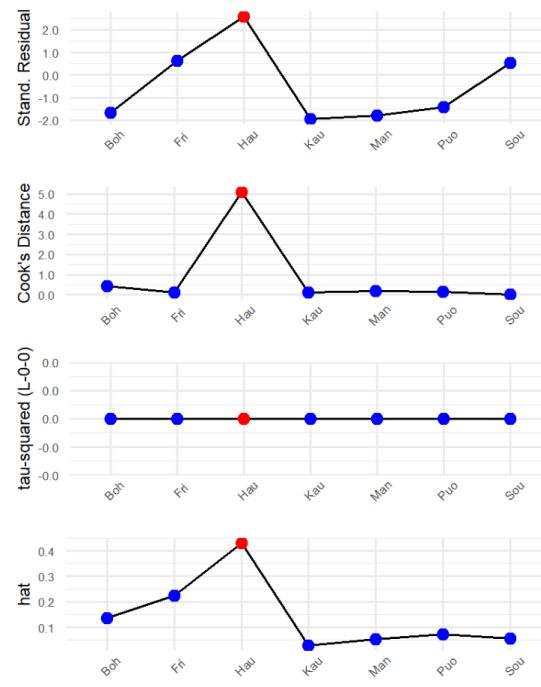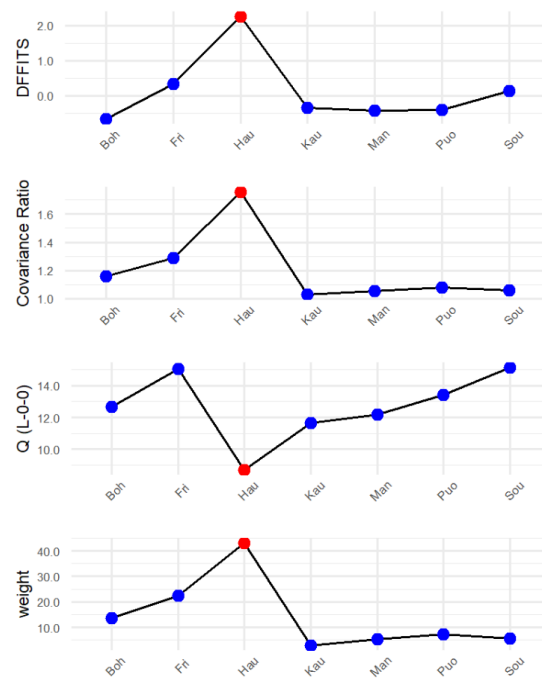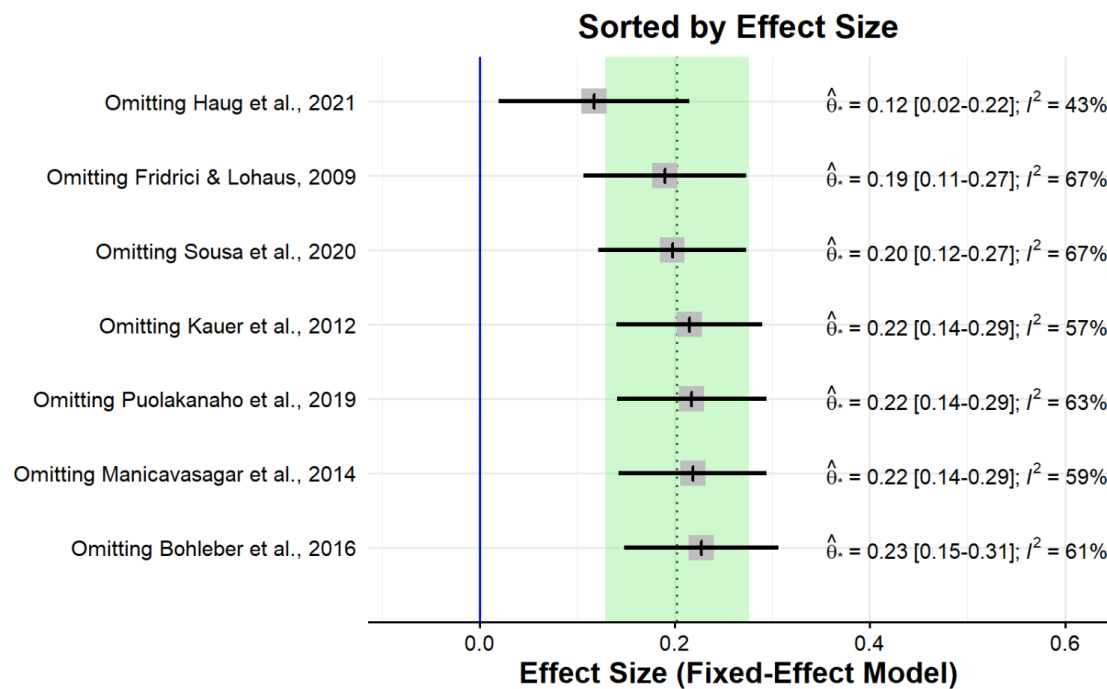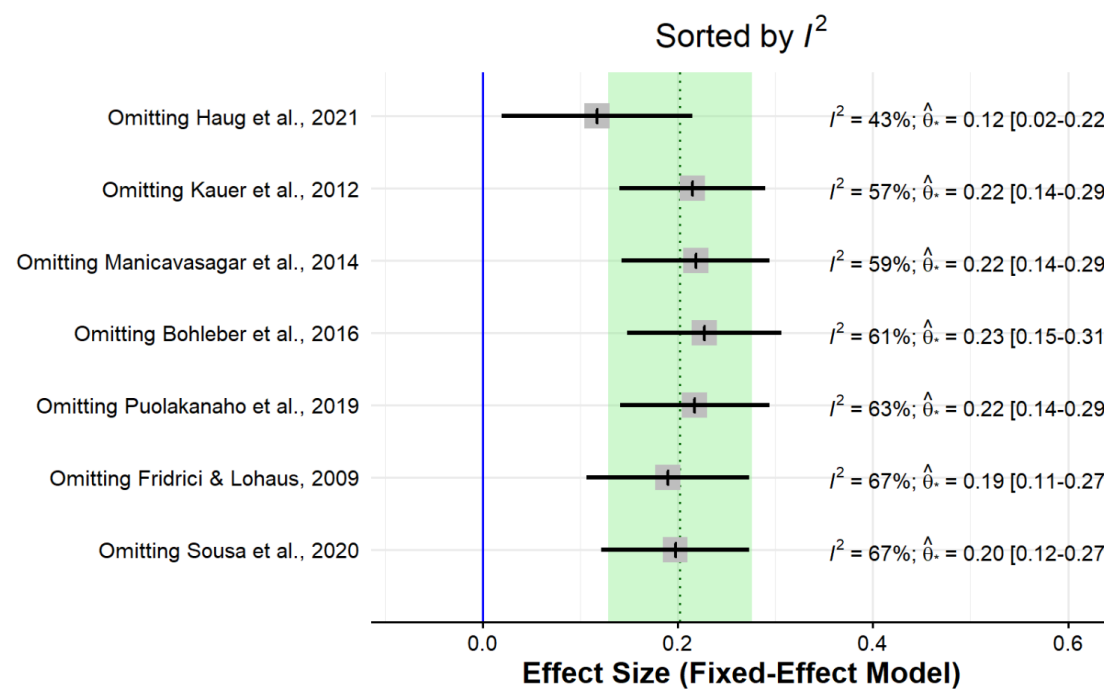

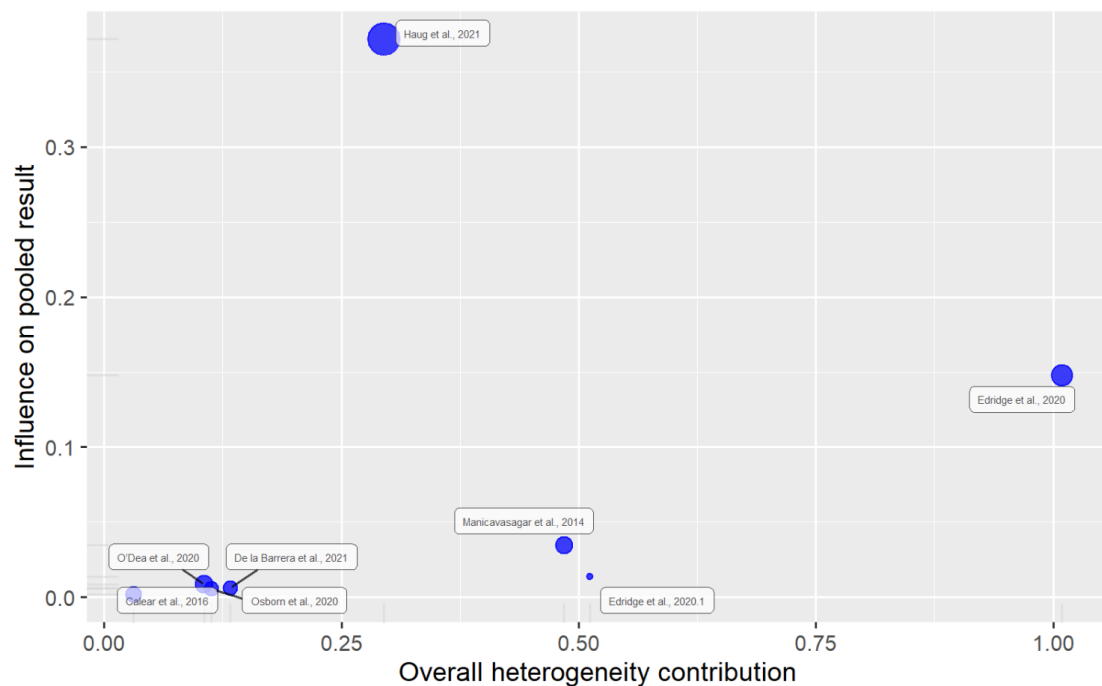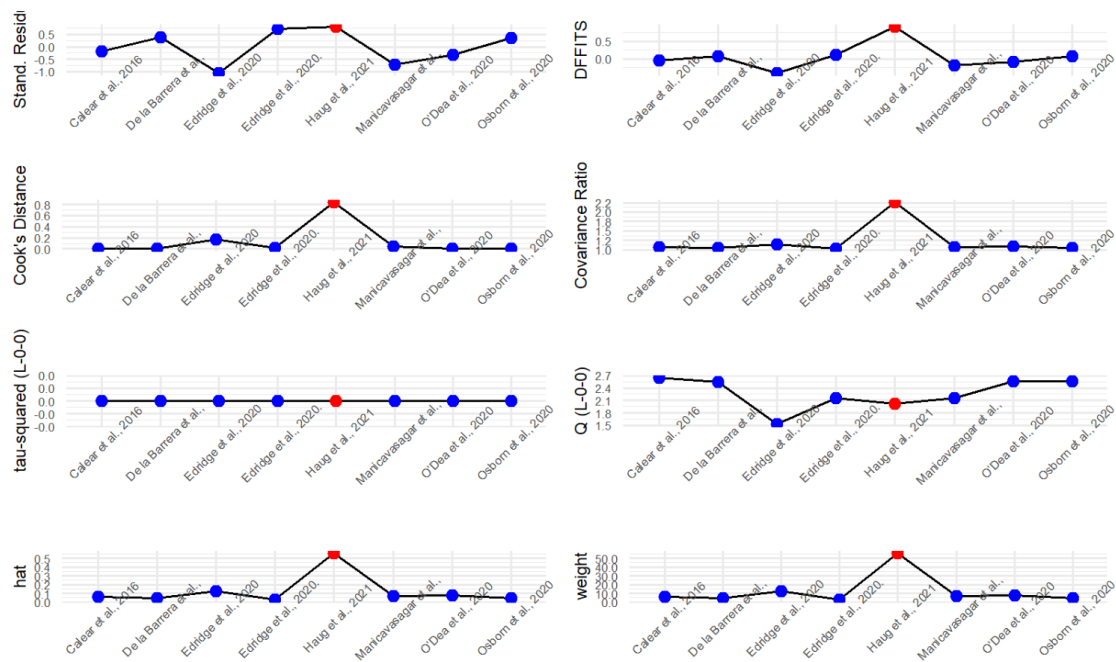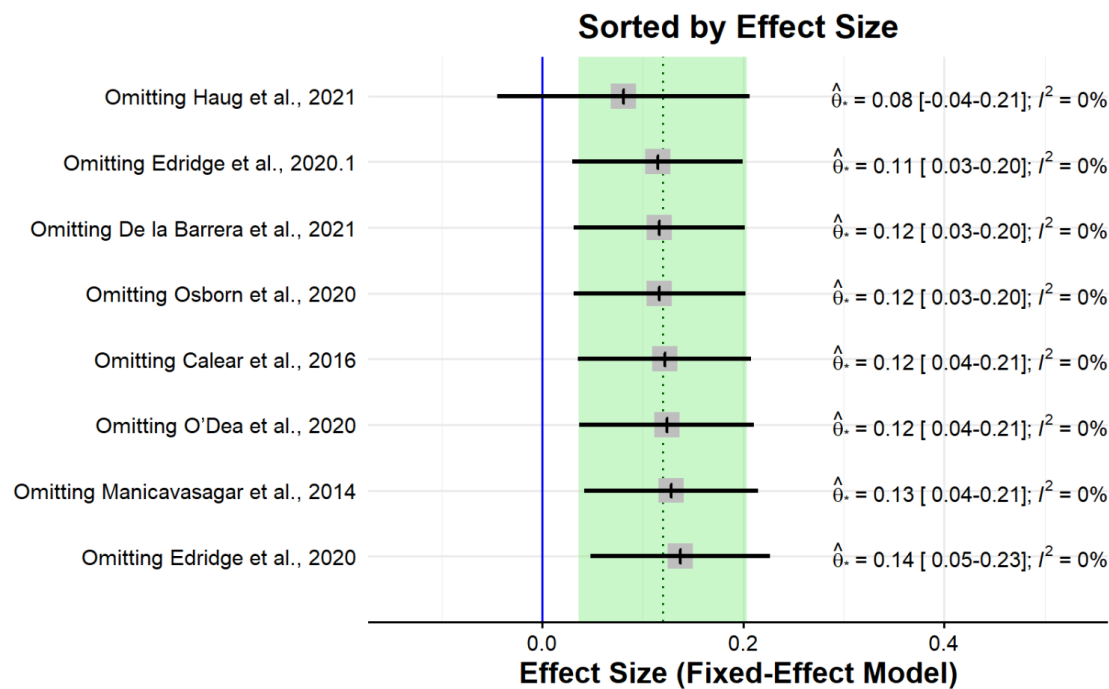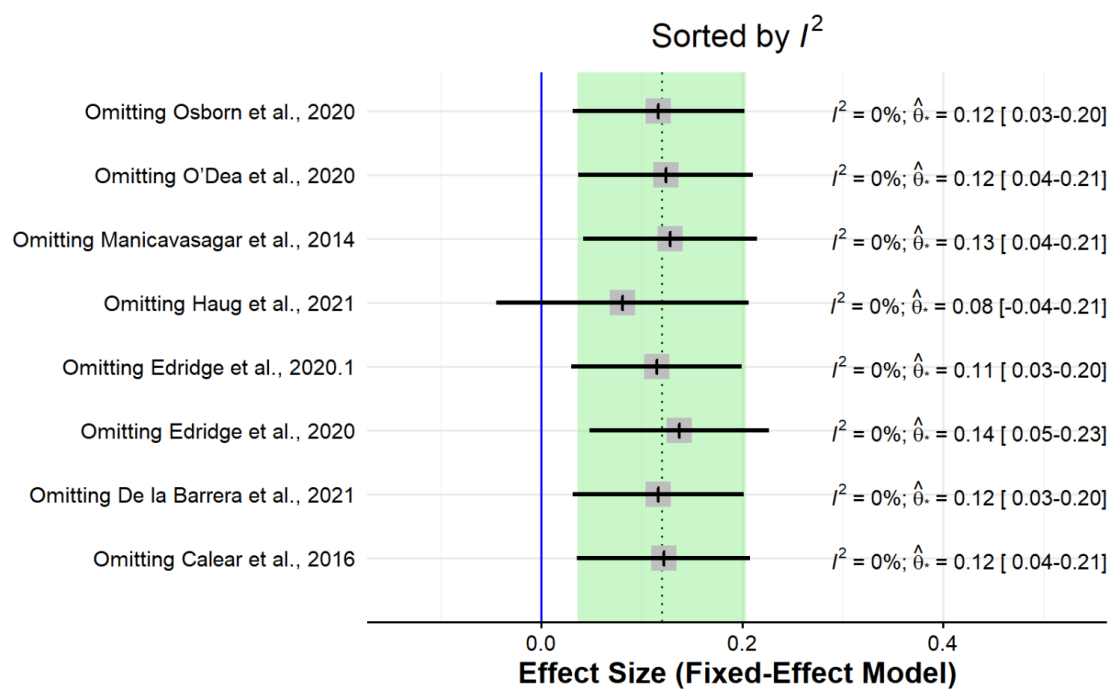

Supplement: Supplementary file 5 — Supplementary Material 5 [file 10964_2023_1735_MOESM5_ESM.pdf]
