# Supplementary material for: Interventions with Digital Tools for Mental Health Promotion among 11–18 Year Olds: A Systematic Review and Meta-Analysis
Source: J Youth Adolesc. 2023 Feb 8;52(4):754–79. doi: 10.1007/s10964-023-01735-4 (PMC9907880; doi:10.1007/s10964-023-01735-4)
Supplement: Supplementary file 4 — Supplementary Material 4 [file 10964_2023_1735_MOESM4_ESM.docx]

| **Supplementary Material 4** | | | | | |  |
| --- | --- | --- | --- | --- | --- | --- |
| *Publication bias analysis with Egger’s Test* | | | | | |  |
| Cluster | n/imputed | Intercept/g | 95% CI | t | p | |
| Anxiety | 11 | -1.40 | [-5.33, -2.53] | -0.70 | 0.504 | |
| Depressive symptoms | 11 | 1.10 | [-1.88, -4.089] | 0.72 | 0.489 | |
| Externalizing symptoms | 6 | 1.71 | [-6.76, -10.18] | 0.40 | 0.713 | |
| Internalizing symptoms | 10 | 0.23 | [-2.11, -2.57] | 0.20 | 0.850 | |
| Protective factors | 11 | -0.42 | [-10.72, -9.88] | -0.08 | 0.938 | |
| Stress  *Duval & Tweedie Trim & Fill* | 7  *9* | -2.89  *0.20* | [-4.69, -1.10]  *[0.00, 0.40]* | -3.16  *2.31* | 0.025  *0.049* | |
| Well-being | 8 | -0.13 | [-1.06, -0.81] | -0.26 | 0.802 | |
